# Back-scatter based whispering gallery mode sensing

**DOI:** 10.1038/srep02974

**Published:** 2013-10-17

**Authors:** Joachim Knittel, Jon D. Swaim, David L. McAuslan, George A. Brawley, Warwick P. Bowen

**Affiliations:** 1 School of Mathematics and Physics, University of Queensland, St Lucia, QLD 4072, Australia; 2 Centre for Engineered Quantum Systems, School of Mathematics and Physics, University of Queensland, St Lucia, Brisbane, QLD 4072, Australia

## Abstract

Whispering gallery mode biosensors allow selective unlabelled detection of single proteins and, combined with quantum limited sensitivity, the possibility for noninvasive real-time observation of motor molecule motion. However, to date technical noise sources, most particularly low frequency laser noise, have constrained such applications. Here we introduce a new technique for whispering gallery mode sensing based on direct detection of back-scattered light. This experimentally straightforward technique is immune to frequency noise in principle, and further, acts to suppress thermorefractive noise. We demonstrate 27 dB of frequency noise suppression, eliminating frequency noise as a source of sensitivity degradation and allowing an absolute frequency shift sensitivity of 76 kHz. Our results open a new pathway towards single molecule biophysics experiments and ultrasensitive biosensors.

Whispering gallery mode (WGM) biosensors combine high quality optical resonators with surface functionalisation to allow ultra-sensitive detection of unlabelled biomolecules such as proteins and nucleic acids^1^,^2^. When a biomolecule enters the evanescent field of the sensor, it becomes polarised and the sensor's optical resonance experiences a reactive shift to lower frequency^3^. This frequency shift can be directly measured with high precision using the optical field exiting the resonator. Operation at the fundamental noise limit dictated by the combination of quantum shot noise and thermo-refractive noise could in principle enable single binding events of unlabelled proteins to be easily resolved, and real-time observation of conformational changes and motor molecule motion^4^. These capacities have significance for many applications in fundamental science, industry, and medical diagnosis. However, such applications have not been possible to date, with technical noise constraining all WGM biosensors to sensitivities many orders of magnitude worse than the fundamental limit. Several avenues have been developed to increase the single-molecule frequency shift and thereby elude technical noise sources, including most notably the use of electric field hot-spots created by plasmonic nanoparticles deposited on the resonator^5^,^6^,^7^,^8^ and of thermo-optic heating of the resonator by the biomolecule itself^9^. In both of these cases, single molecule sensitivity has been achieved^8^. However, similarly to more conventional reactive sensing, the sensitivity of these experiments is dominated by laser frequency noise and remains orders of magnitude above the fundamental limit.

Over the past few years significant efforts have been made to reduce technical noise sources, and most particularly laser frequency noise. In a notable example, direct laser frequency noise cancellation has been achieved using an independent reference interferometer to characterise the laser frequency noise in real-time as experiments proceed^10^. This allowed the detection of 12.5 nm radius nanoparticles with a frequency shift noise floor of 133 kHz. Self-referencing provides an elegant alternative approach to suppress frequency noise, and is particularly well suited to WGM sensors since they inherently exhibit frequency degenerate pairs of forwards- and backwards-propagating optical modes. The presence of a nanoparticle or biomolecule introduces scattering between these modes, breaking the degeneracy and causing a frequency split pair of orthogonal standing wave modes to form in the resonator^11^. Measuring the splitting then provides a self-referenced method to sense the presence of the scatterer that is in principle frequency noise immune. This approach was first demonstrated in Ref. 12, where nanoparticles of 30 nm radius were detected in air by scanning laser spectroscopy of the frequency splitting. However, such spectroscopic techniques are constrained to frequency splittings larger than the resonator optical decay rate, where the splitting becomes spectroscopically resolvable. In the best WGM biosensing experiments to date, the optical decay rate has been in the range of a few MHz^9^ precluding the possibility of detecting single proteins or other small molecules. Proof-of-principle experiments in air have recently overcome this constraint both by embedding the resonator within a Sagnac interferometer and thereby allowing independent measurements of the frequency of each standing wave mode^13^; and by doping the resonator material with a laser medium, pumping it such that both modes exhibit lasing, and directly measuring the beat frequency between them^11^. To the authors knowledge, the latter of these experiments achieved the lowest frequency noise floor previously reported in any WGM sensor of 100 kHz. However, both of these approaches involve a substantial increase in complexity over conventional methods which restricts practical applications.

Here, we report a new approach to self-referenced real-time WGM biosensing that can be straightforwardly applied within conventional sensing configurations. The essence of the approach is to directly monitor the intensity of back-scattered light from the resonator. By scattering light from the forwards- to the backwards-propagating resonator modes, the presence of a biomolecule or nanoparticle directly affects this intensity. We show theoretically that this approach is immune to laser frequency noise in principle; and that, when compared to a direct frequency shift measurements on a single cavity mode, achieves the same quantum shot noise sensitivity limit, with thermorefractive noise suppressed by a factor of two. A proof-of-principle experiment is implemented using an atomic force microscopy (AFM) tip to controllably mimic the presence of a biomolecule^14^. Twenty seven dB of frequency noise suppression is achieved, eliminating frequency noise as a limiting factor for the sensitivity of the sensor. This compares to 20 dB reported in Ref. 10. In that work, however, a large external interferometer was required which was thermally and mechanically insulated and stabilised for several hours in an ice-water bath. By contrast, in our work, no additional optical or electronic systems are required. The absolute frequency noise floor achieved in our experiments is 76 kHz, which to our knowledge is superior to all previous WGM sensors^11^. Furthermore, the approach is directly compatible with enhanced WGM sensing approaches using both plasmonic field hot-spots^5^,^6^,^7^,^8^ and thermo-optic heating^9^.

## Results

### Theoretical model

**Figure 1:**
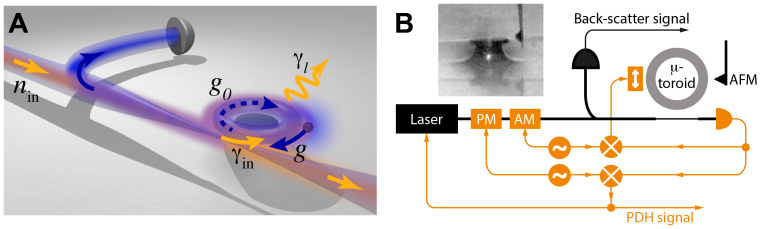
Back-scatter based WGM biosensor. (A): Illustration of concept. Resonator loss rate: *γ_l_* = *γ* − *γ*_in_. (B): Schematic of experiment. Black components: critical components for back-scatter measurement. Orange components: components used for locking and direct frequency measurement. Inset: optical micrograph of sensor. PM: Phase modulator. AM: Amplitude modulator. Orange box containing vertical double-sided arrow: nano-positioning stage used to control separation of taper and toroid.

As shown in Fig. 1A, we consider a WGM resonator with total decay rate *γ*, coupled to an external optical field at rate *γ*_in_. The optical field is detuned a frequency Δ from the optical resonance frequency. An intrinsic scattering rate *g*_0_ is included between the forwards- and backwards-propagating whispering gallery modes, which in experiments typically results from surface roughness and Rayleigh scattering. The backscattered field arise both from this intrinsic scatter and from scattering introduced by the possible presence of a biomolecule or nanoparticle. The frequency shift experienced by the standing-wave mode that interacts with the biomolecule or nanoparticle is exactly equal to twice the scattering rate *g* it introduces between counter-propagating modes^12^. Consequently, a measurement of the scattered intensity directly yields information about the the frequency separation of the split standing wave modes and thereby the polarisability of the scatterer. In the Supplementary Information we calculate the frequency noise spectrum *S*(*ω*) of a measurement of *g* in the realistic limit that 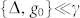 and 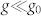, with the result being 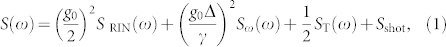
where 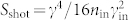 is the quantum shot noise floor of the measurement, with *n*_in_ being the incident photon flux; *S_T_*(*ω*) is the thermorefractive noise floor for measurements on one circulating WGM mode; *S*_RIN_(*ω*) is the power spectrum of relative intensity noise of the incident laser; and *S_ω_*(*ω*) is the power spectrum of the laser frequency noise. As can be seen, for zero detuning (Δ = 0) laser frequency noise is perfectly suppressed. This is in distinct contrast to conventional approaches to WGM biosensing that rely on direct measurements of resonant frequency shifts. In that case, laser frequency noise is indistinguishable from signals due to the presence of the biomolecule or nanoparticle (see Supplementary Information).

When Δ = 0 the remaining noise in Eq. (1) above is due only to quantum shot noise, laser relative intensity noise, and thermorefractive noise. Somewhat remarkably, the quantum shot noise contribution is exactly identical to that of an ideal conventional measurement, while the thermorefractive noise is suppressed by a factor of two (see Supplementary Information for an explicit comparison). This thermorefractive noise suppression can be understood since in the back-scatter configuration the thermorefractive noise arises from back-scattering due to thermally driven local variations in refractive index. This causes a combination of both amplitude and phase fluctuations in the total back-scattered field, with the ratio dependent on the spatial origin of the thermorefractive back-scattering. Averaged over all possible origins within the WGM resonator, the fluctuations are imprinted equally on amplitude and phase. However, only the amplitude fluctuations contribute to the noise floor. The Supplementary information also considers noise due to input coupling fluctuations, arising for example from variations in the distance between the resonator and the coupling device. To first order, this noise is perfectly suppressed in the usual operating regime of critical coupling, where the incident optical field is fully coupled into the resonator.

### Experimental implementation

To test the predictions of the model, we implemented the experiment shown in Fig. 1B (for details see Methods). The WGM biosensor consisted of a silicon chip based microtoroidal resonator excited on optical resonance (Δ = 0) via a tapered optical fibre. The back-scatter signal was extracted from a direct measurement of the backwards-going intensity. This was compared to conventional frequency shift measurements obtained from the transmitted field via a Pound-Drever-Hall (PDH) error signal^15^.

**Figure 2:**
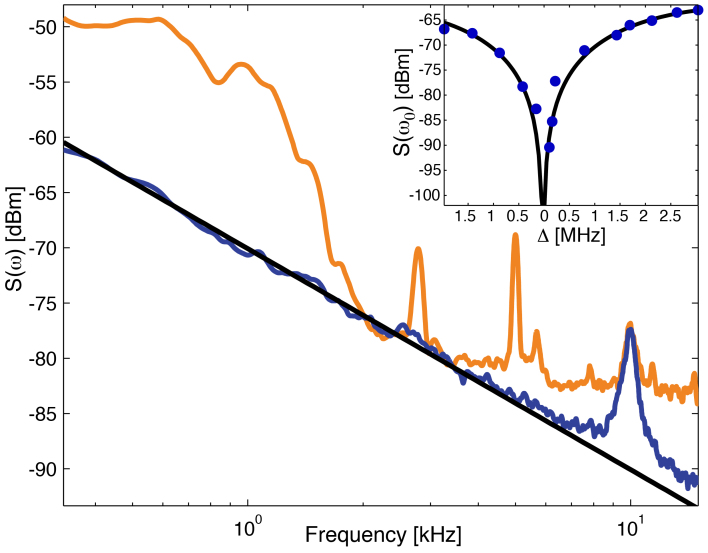
Frequency noise comparison. Frequency noise spectra for back-scatter based (blue/dark trace) and PDH based (orange/light) measurements. Black line: 1/*f*^2^ fit to back-scatter data. Inset: Back-scattered power spectrum at modulation frequency as a function of detuning. Black curve: fit to theory in supplementary information. *ω_0_* : 16 kHz modulation frequency.

In an initial experiment, the relative frequency noise spectra for backscatter and direct frequency shift measurement were obtained using spectral analysis of the back-scattered intensity and the PDH error-signal. The presence of a nanoparticle or biomolecule was controllably simulated by using a nano-positioning stage to place a standard AFM tip within the evanescent field of the toroid. A modulation applied to the position of the AFM tip at it's fundamental resonance frequency of 9.7 kHz caused a sinusoidal variation in both backscattering rate and frequency shift. By equating the power spectra from the two measurements at this frequency, the relative noise floors of the two approaches could be calibrated. The resulting calibrated spectra are shown in Fig. 2. As can be seen, the noise floor of the back-scatter measurement is lower than direct measurement over the full frequency window, and particularly at the low frequencies relevant to biosensing experiments where laser frequency noise becomes increasingly significant. The Lorentzian peak evident at 9.7 kHz in both spectra is the AFMs thermally excited fundamental mechanical mode. With the exception of this peak, the back-scatter measurement is dominated by 1/*f*^2^ noise with no other technical noise evident. We believe that this noise is laser intensity noise, with separate measurements of the laser source revealing the same 1/*f*^2^ dependence. In future experiments it may be possible to greatly suppress the effect of laser intensity noise by either employing a noise eater to directly reduce it, or measuring it simultaneously with the back-scatter measurements and subtracting it in post-processing.

To determine the frequency noise suppression achieved by the back-scatter technique, we apply a 16 kHz modulation to the laser frequency via it's internal PZT, and characterise the power spectrum at this frequency as a function of laser detuning Δ as shown in the inset of Fig. 2. As expected, the observed signal is minimised at zero detuning, with 27 dB of frequency noise suppression evident compared to the far detuned case. With this level of suppression the laser frequency noise, evident as the structural features in the PDH error-signal trace in Fig. 2, lies more than 10 dB beneath the 1/*f*^2^ noise on the back-scatter measurement, and is therefore negligible. The suppression achieved here compares favourably to the 20 dB of suppression reported in Ref. 10 using a cooled and stabilised reference interferometer.

**Figure 3:**
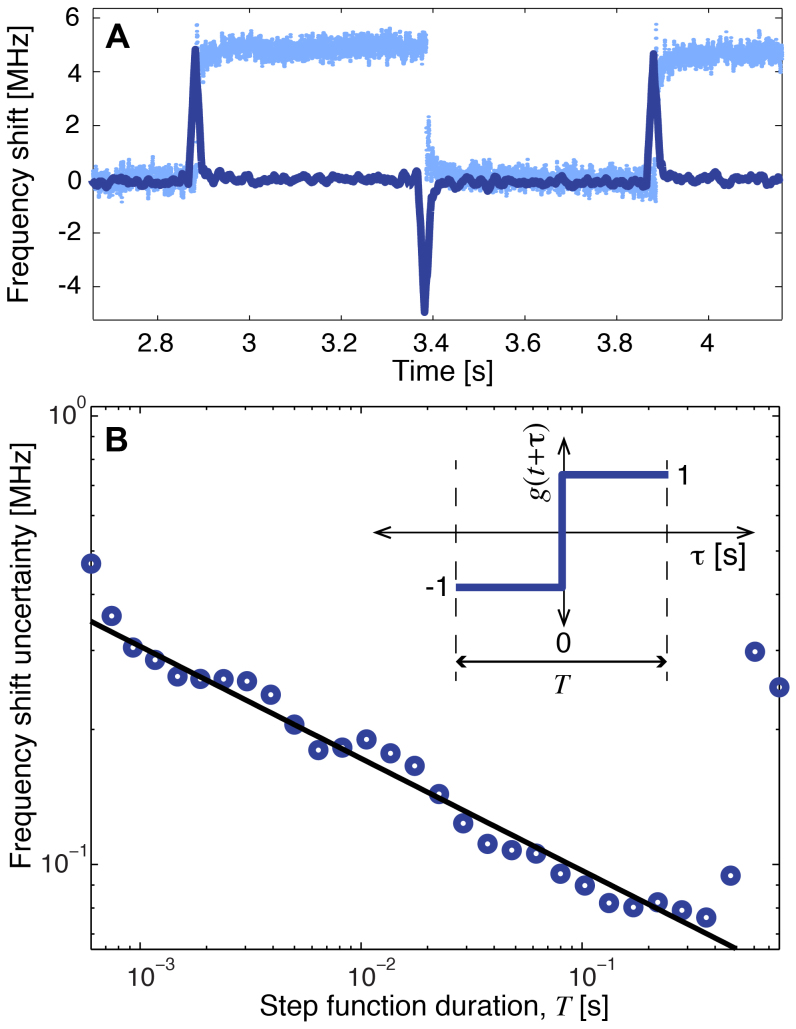
Absolute noise floor of back-scatter measurement. (A): Frequency shift as function of time. Light blue curve: raw frequency shift data *ν*(*t*). Dark blue curve: instantaneous frequency shift *δν*(*t*) obtained using a step function of duration *T* = 30 ms. (B): Frequency shift uncertainty as a function of step function duration. Black line: fit to frequency shift uncertainty with function Δ*ν* = *At*^−1/4^, *A* = 54 kHz s^1/4^. Inset: step function *g*(*t* + *τ*) used to perform cross-correlation.

To demonstrate the absolute noise performance of the back-scatter measurement, a second experiment was conducted, where a square-wave was applied to the AFM position, simulating regular binding events of nanoparticles or biomolecules. As expected, the back-scattered intensity displayed the same square-wave feature (shown in Fig. 3A). A two step process was performed to calibrate the back-scattered intensity in terms of whispering gallery mode resonance frequency shift. First, the response of the laser frequency to a voltage applied to its internal PZT was characterised. The PDH error signal fed back to the laser then provided the frequency shift of the optical mode as a function of the position of the AFM tip, which could be related back to the observed back-scattered intensity. As can be seen in Fig. 3A, when present, the AFM caused a 5 MHz frequency shift, with raw frequency noise at a level of 400 kHz. It is important to note that this raw frequency noise depends greatly on the choice of filtering in the data acquisition and should not be treated with real significance.

To determine the frequency noise floor in a meaningful way a cross-correlation was performed between the raw data and a step function. The time-domain filter 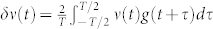 was applied to the raw frequency shift data *ν*(*t*) obtained from the back-scatter measurement, where *g*(*t* + *τ*) is the step-function shown in the inset of Fig. 3B and *T* is its duration. As shown in Fig. 3A (dark blue trace), this converts the frequency shift at time *t* from the total cumulative frequency shift experienced prior to that time (light blue trace), to the frequency shift *δν*(*t*) due to a binding event at time *t* given that no other binding events occur within the time window *t* ± *T*/2. The standard deviation of *δν*(*t*) over the time window where the AFM is stationary provides the frequency noise floor of the measurement. This noise floor depends on the total duration of the step function, improving as the duration increases and more data is included in the cross-correlation, as shown in Fig. 3B. A minimum noise floor of 76 kHz is obtained when the duration of the step function is just under 0.5 s, half the period of the square-wave. A dramatic and spurious degradation in the noise floor is evident once the duration exceeds this time and begins to sample the next simulated binding event. Note that in a real scenario involving uncontrolled detection of nanoparticles or biomolecules, the optimal duration of the step function would be dictated by the magnitude of low frequency noise.

## Discussion

In conclusion, we have proposed and demonstrated a new form of self-referenced whispering gallery mode sensing. Unlike other approaches the technique both allows sensing of frequency shifts smaller than the optical linewidth, and does not require any sophisticated characterisation techniques. Rather, it simply requires direct detection of the back-scattered intensity from a conventional whispering gallery mode sensor. We show theoretically, that the quantum shot noise floor of this approach is equal to that achieved from frequency shift measurement of an equivalent single mode optical cavity, and that the thermorefractive noise limit is halved. The technique is applicable not only to conventional whispering gallery mode sensing configurations, but also to enhanced configurations relying on plasmonic hot-spots or the thermo-optic effect.

Using an AFM tip to simulate biomolecule binding events, we demonstrate that the technique suppresses laser frequency noise by 27 dB, and out-completes direct frequency shift measurements over the frequency band relevant to biosensing. An absolute frequency shift sensitivity of 76 kHz is achieved, to our knowledge surpassing all previous whispering gallery mode sensors^11^. It should be noted that Ref. 11 reported a raw sensitivity of 100 kHz without using the cross-correlation analysis method reported here. Applying this method in their experiments could potentially enable a significant enhancement in sensitivity.

During review of this article we became aware of parallel efforts reported very recently in a conference abstract^17^. These efforts apply the principle of back-scatter based sensing to detect nanoparticles as small as 20 nm in radius and extend work reported in a PhD dissertation^18^. No conclusions are drawn about the fundamental noise limits of the technique or its resistance to laser frequency noise.

## Methods

The microtoroidal resonator had an optical quality factor of approximately 2 × 10^7^, and was optically excited with 4.3 *μ*W of 1557.5 nm laser light. A Thorlabs 6015-3-APC circulator placed in the fibre line prior to the resonator allowed the backscattered intensity to be measured on a New Focus 2011 photoreceiver, with approximately 50 nW of backscattered power observed when the laser was on resonance. The Pound-Drever-Hall (PDH) error signal used to compare back-scatter measurements to conventional frequency shift measurements additionally allowed the laser, a New Focus Velocity external cavity diode laser, to be locked onto the optical resonance frequency via feedback to its internal PZT, enforcing the zero-detuning condition Δ = 0. To further stabilise the experiments, the optical coupling rate to the resonator was locked to critical coupling by amplitude modulating the laser to extract a zero-crossing error signal, and feeding this back to a PZT controlling the position of the tapered optical fibre^16^.

## Author Contributions

J.K. performed the primary experiments with contributions from J.D.S. and G.A.B. D.L.M. performed the laser noise measurements. J.K., G.A.B. and W.P.B. analysed the data. W.P.B. conceived the idea and did the theory. W.P.B. prepared the manuscript. All authors reviewed the manuscript.

## Supplementary Material

Supplementary Information Supplementary information: Back-scatter based whispering gallery mode sensing
