# Supplementary material for: Back-scatter based whispering gallery mode sensing
Source: Sci Rep. 2013 Oct 17;3:2974. doi: 10.1038/srep02974 (PMC3797986; doi:10.1038/srep02974)
Supplement: Supplementary Information — Back-scatter based whispering gallery mode sensing [file srep02974-s1.pdf]

Supplementary information: Back-scatter based whispering gallery mode sensing

Joachim Knittel,[1] Jon D. Swaim,[1] David L. McAuslan,[2] George A. Brawley,[2] and Warwick P. Bowen[2]

[1]School of Mathematics and Physics, University of Queensland, St Lucia, QLD 4072, Australia
[2]Centre for Engineered Quantum Systems, School of Mathematics and Physics, University of Queensland, St Lucia, Brisbane, QLD 4072, Australia

(Dated: August 9, 2013)

Here we derive the sensitivity of back-scatter based whispering gallery mode sensing, and compare it directly to typical dispersive sensing with an optical cavity. The quantum shot noise limit of the two kinds of sensing is found to be identical. However, in contrast to typical dispersive sensing, back-scatter sensing is found to be, in principle, immune to frequency noise. Furthermore, the thermorefractive noise is found to be exactly half that of typical sensing.

A BRIEF EXPLANATION OF THE APPROACH

In what follows we use a standard quantum optics approach to determining the sensitivity limits of WGM sensors (see Ref. [1] for an overview of the method). This approach is nice since it naturally includes, in a formal way, the shot noise due to quantisation of the optical field. However, the approach can be understood classically. Since the entire system is linear, an equivalent result would be obtained by neglecting quantum noise terms (terms like $\delta\tilde{a}$), thinking of $\alpha_j = \langle a_j \rangle$ as being the appropriately scaled electric field amplitude in mode j, and adding shot noise phenomenologically at the end of the derivation.

In the derivation we make the approximation that the sideband frequency ω, total scattering rate between counter propagating modes $g_0 + g_{\text{sig}}$, cavity detuning Δ, and thermorefractive frequency noise are all small compared to the optical linewidth γ. This approximation is accurate for the vast majority of WGM biosensing experiments in water since the optical linewidth is typically above 10 MHz, and each of the terms listed above are typically less that 1 MHz. The theory could, in principle, quite easily be derived without these approximations. However, the mathematics would become much more complex and the end result less enlightening.

DERIVING THE HAMILTONIAN

Consider two counter-propagating degenerate WGM resonances, a clock-wise mode (C) and anti-clockwise mode (A), with respective modeshapes $U_C(\mathbf{r}) = V(r,\theta)e^{im\phi}$ and $U_A(\mathbf{r}) = V(r,\theta)e^{-im\phi}$, where r, θ, and ϕ are the usual cylindrical co-ordinates, and m is the azimuthal mode number. The bare Hamiltonian of the system is

$$H_0 = \hbar\Omega\left(\hat{a}_A^\dagger\hat{a}_A + \hat{a}_C^\dagger\hat{a}_C\right) \tag{1}$$

where we have neglected the constant term due to the zero-point energy of the system.

The total Hamiltonian of the system contains both the bare Hamiltonian and terms $\mathcal{V}$ due to the energy of polarizable particles within the electric field $\hat{E}$ each mode. Defining the modeshapes to be normalised we have $\int |U_A|^2 d\mathbf{V} = \int |U_C|^2 d\mathbf{V} = 1$; and since $\int |e^{\pm im\phi}|^2 d\phi = 2\pi$, $\int |V(r,\theta)|^2 dr d\theta = 1/2\pi$. Assuming no other resonant modes are nearby, the electric field within the WGM resonator can be approximated by a sum of the electric fields of each of these modes

$$\hat{E} = \mathcal{E}e^{i\Omega t}\left(\hat{a}_A^\dagger U_A^*(\mathbf{r}) + \hat{a}_C^\dagger U_C^*(\mathbf{r})\right) + h.c. \tag{2}$$

$$= \mathcal{E}V^*(r,\theta)e^{i\Omega t}\left(\hat{a}_A^\dagger e^{im\phi} + \hat{a}_C^\dagger e^{-im\phi}\right) + h.c. \tag{3}$$

where $\hat{a}_j$ is the annihilation operator of mode j normalised such that $\langle \hat{a}_j^\dagger \hat{a}_j \rangle = \langle \hat{n}_j \rangle$ and $[\hat{a}_j, \hat{a}_j^\dagger] = 1$, and $\mathcal{E}$ is the quantised field strength (i.e. the zero point electric field magnitude in each mode)

$$\mathcal{E} = \sqrt{\frac{\hbar\Omega}{2\epsilon_0 V}}, \tag{4}$$

where, since the two modes are symmetric apart from propagation direction, with degenerate frequency and volume, their quantised field strengths are identical.

We wish to consider microscopic fluctuations in the polarizability of the WGM structure. Therefore, we introduce the polarizability density $\rho_\alpha(\mathbf{r})$ which describes the capacity of an electric field to induce a change in polarization around the point $\mathbf{r}$. The polarization density is then just

$$\rho_p(\mathrm{r}) = \epsilon_0 \rho_\alpha(\mathbf{r})\hat{E}(\mathbf{r}) \tag{5}$$

$$= \epsilon_0 \mathcal{E} \rho_\alpha(\mathbf{r}) \left[V^*(r,\theta) e^{i\Omega t}\left(\hat{a}_A^\dagger e^{im\phi} + \hat{a}_C^\dagger e^{-im\phi}\right) + V(r,\theta)e^{-i\Omega t}\left(\hat{a}_A e^{-im\phi} + \hat{a}_C e^{im\phi}\right)\right]. \tag{6}$$

Since for a single dipole, the polarization energy is $\mathcal{V} = -\mathrm{p}\cdot\hat{E} = -p\hat{E}$, the polarization energy density is

$$\rho_{\mathcal{V}}(\mathrm{r}) = -\rho_p(\mathbf{r})\hat{E} \tag{7}$$

$$= -\epsilon_0 \mathcal{E}^2 \rho_\alpha(\mathbf{r}) \left[V^*(r,\theta) e^{i\Omega t}\left(\hat{a}_A^\dagger e^{im\phi} + \hat{a}_C^\dagger e^{-im\phi}\right) + V(r,\theta)e^{-i\Omega t}\left(\hat{a}_A e^{-im\phi} + \hat{a}_C e^{im\phi}\right)\right]^2 \tag{8}$$

$$= -\epsilon_0 \mathcal{E}^2 \rho_\alpha(\mathbf{r}) |V(r,\theta)|^2 \left[\hat{a}_A^\dagger \hat{a}_A + \hat{a}_C^\dagger \hat{a}_C + \hat{a}_A^\dagger \hat{a}_C e^{2im\phi} + \hat{a}_A \hat{a}_C^\dagger e^{-2im\phi}\right]. \tag{9}$$

Where we've made the usual rotating wave approximation, neglecting fast oscillating terms in time. The polarization energy is then

$$\mathcal{V} = \int \rho_{\mathcal{V}}(\mathrm{r}) d\mathbf{V} \tag{10}$$

$$= -\epsilon_0 \mathcal{E}^2 \left[\int \rho_\alpha(\mathbf{r})|V(r,\theta)|^2 d\mathbf{V}\left(\hat{a}_A^\dagger \hat{a}_A + \hat{a}_C^\dagger \hat{a}_C\right) + \int \rho_\alpha(\mathbf{r})|V(r,\theta)|^2 e^{2im\phi} d\mathbf{V} \hat{a}_A^\dagger \hat{a}_C + \int \rho_\alpha(\mathbf{r})|V(r,\theta)|^2 e^{-2im\phi} d\mathbf{V} \hat{a}_A \hat{a}_C^\dagger\right] \tag{11}$$

$$= -\hbar g_{\mathrm{self}}\left(\hat{a}_A^\dagger \hat{a}_A + \hat{a}_C^\dagger \hat{a}_C\right) - \hbar g_{\mathrm{cross}} \hat{a}_A^\dagger \hat{a}_C - \hbar g_{\mathrm{cross}}^* \hat{a}_A \hat{a}_C^\dagger \tag{12}$$

where

$$\hbar g_{\mathrm{self}} = \epsilon_0 \mathcal{E}^2 \int \rho_\alpha(\mathbf{r})|V(r,\theta)|^2 d\mathbf{V} \tag{13}$$

$$\hbar g_{\mathrm{cross}} = \epsilon_0 \mathcal{E}^2 \int \rho_\alpha(\mathbf{r})|V(r,\theta)|^2 e^{2im\phi} d\mathbf{V}, \tag{14}$$

respectively represent the polarization induced self-energy of modes A and C, the interaction energy between them.

We consider three different contributions to the polarizability density: the nanoparticle that we seek to detect with point-like polarizability α_{sig} located at $\mathbf{r}_{\mathrm{sig}}$, a static defect scattering center on the WGM with point-like polarizability α_0 located at $\mathbf{r}_0$, and a spatially and temporally fluctuating polarizability density $\rho_{\alpha,\mathrm{therm}}(\mathrm{r}, t)$. The total polarizability density is then

$$\rho_\alpha(\mathbf{r}) = \alpha_{\mathrm{sig}}\delta(\mathbf{r}_{\mathrm{sig}}) + \alpha_0 \delta(\mathbf{r}_0) + \rho_{\alpha,\mathrm{therm}}(\mathbf{r}, t). \tag{15}$$

Substituting this into Eq. (13) above we find

$$\hbar g_{\mathrm{self}} = \epsilon_0 \mathcal{E}^2 \int \left(\alpha_{\mathrm{sig}}\delta(\mathbf{r}_{\mathrm{sig}}) + \alpha_0\delta(\mathbf{r}_0) + \rho_{\alpha,\mathrm{therm}}(\mathbf{r},t)\right)|V(r,\theta)|^2 d\mathbf{V} \tag{16}$$

$$= \epsilon_0 \mathcal{E}^2 \left(\alpha_{\mathrm{sig}}|V(r_{\mathrm{sig}},\theta_{\mathrm{sig}})|^2 + \alpha_0 |V(r_0,\theta_0)|^2 + \int \rho_{\alpha,\mathrm{therm}}(\mathbf{r},t)|V(r,\theta)|^2 d\mathbf{V}\right) \tag{17}$$

$$= \hbar\left(g_{\mathrm{sig}} + g_0 + g_{\mathrm{therm}}^{\mathrm{self}}\right) \tag{18}$$

Similarly, substituting into Eq. (14) we find

$$\hbar g_{\mathrm{cross}} = \epsilon_0 \mathcal{E}^2 \int \left(\alpha_{\mathrm{sig}}\delta(\mathbf{r}_{\mathrm{sig}}) + \alpha_0\delta(\mathbf{r}_0) + \rho_{\alpha,\mathrm{therm}}(\mathbf{r},t)\right)|V(r,\theta)|^2 e^{2im\phi} d\mathbf{V} \tag{19}$$

$$= \epsilon_0 \mathcal{E}^2 \left(\alpha_{\mathrm{sig}}|V(r_{\mathrm{sig}},\theta_{\mathrm{sig}})|^2 e^{2im\phi_{\mathrm{sig}}} + \alpha_0 |V(r_0,\theta_0)|^2 e^{2im\phi_0} + \int \rho_{\alpha,\mathrm{therm}}(\mathbf{r},t)|V(r,\theta)|^2 e^{2im\phi} d\mathbf{V}\right) \tag{20}$$

$$= \hbar\left(g_{\mathrm{sig}} e^{2im\phi_{\mathrm{sig}}} + g_0 e^{2im\phi_0} + g_{\mathrm{therm}}^{\mathrm{cross}}\right) \tag{21}$$

The modification to the Hamiltonian $\mathcal{V}$ due to polarizable particles is therefore

$$\mathcal{V} = -\hbar\left(g_{\mathrm{sig}} + g_0 + g_{\mathrm{therm}}^{\mathrm{self}}\right)\left(\hat{a}_A^\dagger \hat{a}_A + \hat{a}_C^\dagger \hat{a}_C\right) - \hbar\left(g_{\mathrm{sig}} e^{2im\phi_{\mathrm{sig}}} + g_0 e^{2im\phi_0} + g_{\mathrm{therm}}^{\mathrm{cross}}\right)\hat{a}_A^\dagger \hat{a}_C - \hbar\left(g_{\mathrm{sig}} e^{-2im\phi_{\mathrm{sig}}} + g_0 e^{-2im\phi_0} + g_{\mathrm{therm}}^{\mathrm{cross}\,*}\right)\hat{a}_A \hat{a}_C^\dagger \tag{22}$$

with the total Hamiltonian being

$$H = H_0 + \mathcal{V} \tag{23}$$

We see that each of the nanoparticle, defect centre, and thermorefractive noise introduce both a shift in the WGM resonance frequencies, and a coupling rate between them.

We can simplify the problem, by moving into a rotating frame at frequency $\Omega - \Delta_0$, where Δ_0 is the detuning of the laser field from the bare optical resonance. To do this, we apply the unitary $\hat{U} = e^{-i(\Omega+\Delta_0)t}$ such that $\tilde{H} = \hat{U}^\dagger \hat{H} \hat{U} - \hbar(\Omega - \Delta_0)$; where the $\tilde{}$ denotes throughout a Hamiltonian or operator in the the rotating frame. We then arrive at

$$\tilde{H} = -\hbar\left(g_{\text{sig}} + g^{\text{self}}_{\text{therm}} + \Delta\right)\left(\tilde{a}^\dagger_A\tilde{a}_A + \tilde{a}^\dagger_C\tilde{a}_C\right) - \hbar\left(g_{\text{sig}}e^{2im\phi_{\text{sig}}} + g_0 e^{2im\phi_0} + g^{\text{cross}}_{\text{therm}}\right)\tilde{a}^\dagger_A\tilde{a}_C - \hbar\left(g_{\text{sig}}e^{-2im\phi_{\text{sig}}} + g_0 e^{-2im\phi_0} + g^{\text{cross}\,*}_{\text{therm}}\right)\tilde{a}_A\tilde{a}^\dagger_C, \tag{24}$$

with $\tilde{a} = \hat{a}e^{-i(\Omega-\Delta)t}$, and $\Delta = \Delta_0 + g_0$ being the modified cavity defining due to the scattering centre g_0.

Variances of fluctuating energy terms

The energy terms due to thermorefractive noise $g^{\text{self}}_{\text{therm}}$ and $g^{\text{cross}}_{\text{therm}}$ are both zero-mean noise terms. Later on in this document it will be useful to know the relationship between their variances.

The variance of $g^{\text{self}}_{\text{therm}}$ is

$$\begin{aligned}
\hbar^2\left\langle g^{\text{self}\,2}_{\text{therm}}\right\rangle &= \epsilon_0^2\mathcal{E}^4\left\langle\int\rho_{\alpha,\text{therm}}(\mathbf{r},t)|V(r,\theta)|^2 d\mathbf{V}\int\rho_{\alpha,\text{therm}}(\mathbf{r}',t)|V(r',\theta')|^2 d\mathbf{V}'\right\rangle && (25)\\
&= \epsilon_0^2\mathcal{E}^4\iint\left\langle\rho_{\alpha,\text{therm}}(\mathbf{r},t)\rho_{\alpha,\text{therm}}(\mathbf{r}',t)\right\rangle|V(r,\theta)|^2|V(r',\theta')|^2 d\mathbf{V}d\mathbf{V}' && (26)\\
&= \epsilon_0^2\mathcal{E}^4\int\left\langle\rho_{\alpha,\text{therm}}(\mathbf{r},t)^2\right\rangle|V(r,\theta)|^4 d\mathbf{V} && (27)\\
&= \frac{\epsilon_0^2\mathcal{E}^4}{2\pi}\iint\left\langle\rho_{\alpha,\text{therm}}(r,\theta,t)^2\right\rangle|V(r,\theta)|^4 dr d\theta && (28)
\end{aligned}$$

where we've used the usual approximation that thermorefractive noise is delta-correlated, $\langle\rho_{\alpha,\text{therm}}(\mathbf{r},t)\rho_{\alpha,\text{therm}}(\mathbf{r}',t)\rangle = \langle\rho_{\alpha,\text{therm}}(\mathbf{r},t)^2\rangle\delta_{\text{r=r}'}$, and, assuming a homogeneous material have taken the average thermorefractive noise to have no ϕ dependence.

Unlike $g^{\text{self}}_{\text{therm}}$, $g^{\text{cross}}_{\text{therm}}$ can in general be complex. We therefore break it into real and imaginary parts $g^{\text{cross}}_{\text{therm}} = R^{\text{cross}}_{\text{therm}} + iI^{\text{cross}}_{\text{therm}}$ and calculate the variance of each independently.

$$\begin{aligned}
\hbar R^{\text{cross}}_{\text{therm}} &= \int\rho_{\alpha,\text{therm}}(\mathbf{r},t)|V(r,\theta)|^2\cos 2m\phi d\mathbf{V} && (29)\\
\hbar I^{\text{cross}}_{\text{therm}} &= \int\rho_{\alpha,\text{therm}}(\mathbf{r},t)|V(r,\theta)|^2\sin 2m\phi d\mathbf{V} && (30)
\end{aligned}$$

The real variance is then

$$\begin{aligned}
\hbar^2\left\langle R^{\text{cross}\,2}_{\text{therm}}\right\rangle &= \epsilon_0^2\mathcal{E}^4\left\langle\int\rho_{\alpha,\text{therm}}(\mathbf{r},t)|V(r,\theta)|^2\cos 2m\phi d\mathbf{V}\int\rho_{\alpha,\text{therm}}(\mathbf{r}',t)|V(r',\theta')|^2\cos 2m\phi' d\mathbf{V}'\right\rangle && (31)\\
&= \epsilon_0^2\mathcal{E}^4\iint\left\langle\rho_{\alpha,\text{therm}}(\mathbf{r},t)\rho_{\alpha,\text{therm}}(\mathbf{r}',t)\right\rangle|V(r,\theta)|^2|V(r',\theta')|^2\cos 2m\phi\cos 2m\phi' d\mathbf{V}d\mathbf{V}' && (32)\\
&= \epsilon_0^2\mathcal{E}^4\int\left\langle\rho_{\alpha,\text{therm}}(\mathbf{r},t)^2\right\rangle|V(r,\theta)|^4\cos^2 2m\phi d\mathbf{V} && (33)\\
&= \frac{\epsilon_0^2\mathcal{E}^4}{2}\iint\left\langle\rho_{\alpha,\text{therm}}(\mathbf{r},t)^2\right\rangle|V(r,\theta)|^4 dr d\theta\int 1 + \cos 4m\phi d\phi && (34)\\
&= \frac{\epsilon_0^2\mathcal{E}^4}{4\pi}\iint\left\langle\rho_{\alpha,\text{therm}}(r,\theta,t)^2\right\rangle|V(r,\theta)|^4 dr d\theta && (35)\\
&= \frac{1}{2}\hbar^2\left\langle g^{\text{self}\,2}_{\text{therm}}\right\rangle && (36)
\end{aligned}$$

Similarly for the imaginary term

$$\hbar^2 \left\langle I^{\text{cross } 2}_{\text{therm}} \right\rangle = \epsilon_0^2 \mathcal{E}^4 \left\langle \int \rho_{\alpha,\text{therm}}(\mathbf{r},t) |V(r,\theta)|^2 \sin 2m\phi d\mathbf{V} \int \rho_{\alpha,\text{therm}}(\mathbf{r}',t) |V(r',\theta')|^2 \cos 2m\phi' d\mathbf{V}' \right\rangle \tag{37}$$

$$= \epsilon_0^2 \mathcal{E}^4 \iint \langle \rho_{\alpha,\text{therm}}(\mathbf{r},t) \rho_{\alpha,\text{therm}}(\mathbf{r}',t) \rangle |V(r,\theta)|^2 |V(r',\theta')|^2 \sin 2m\phi \sin 2m\phi' d\mathbf{V} d\mathbf{V}' \tag{38}$$

$$= \epsilon_0^2 \mathcal{E}^4 \int \left\langle \rho_{\alpha,\text{therm}}(\mathbf{r},t)^2 \right\rangle |V(r,\theta)|^4 \sin^2 2m\phi d\mathbf{V} \tag{39}$$

$$= \frac{\epsilon_0^2 \mathcal{E}^4}{2} \iint \left\langle \rho_{\alpha,\text{therm}}(\mathbf{r},t)^2 \right\rangle |V(r,\theta)|^4 dr d\theta \int 1 - \cos 4m\phi d\phi \tag{40}$$

$$= \frac{\epsilon_0^2 \mathcal{E}^4}{4\pi} \iint \left\langle \rho_{\alpha,\text{therm}}(r,\theta,t)^2 \right\rangle |V(r,\theta)|^4 dr d\theta \tag{41}$$

$$= \frac{1}{2} \hbar^2 \left\langle g^{\text{self } 2}_{\text{therm}} \right\rangle \tag{42}$$

Hence we see that

$$\left\langle R^{\text{cross } 2}_{\text{therm}} \right\rangle = \left\langle I^{\text{cross } 2}_{\text{therm}} \right\rangle = \frac{1}{2} \left\langle g^{\text{self } 2}_{\text{therm}} \right\rangle \tag{43}$$

DIRECT MEASUREMENT OF THE PHASE SHIFT ON A SINGLE MODE AS A BENCHMARK

The usual approach to dispersive measurement in an optical resonator is to excite the resonator on resonance, and directly measure the phase shift introduced on the field exiting the resonator. This approach has been shown, in principle, to be optimal, reaching the quantum limit due to shot noise. Here, prior to considering the full measurement protocol, we consider this simplified case to derive the quantum limit on measurements of the signal scattering rate g_{sig} (or equivalently, the polarizability of the nanoparticle). The Hamiltonian is then found by dropping all of the coupling terms from Eq. (24), and considering only one of the two counter-propagating modes, dropping the redundant subscript.

$$\tilde{H}_{\text{QNL}} = -\hbar \left(g_{\text{sig}} + g^{\text{self}}_{\text{therm}} + \Delta \right) \tilde{a}^\dagger \tilde{a} \tag{44}$$

$$= -\hbar \left(g_{\text{sig}} + g^{\text{self}}_{\text{therm}} + \Delta \right) \tilde{a}^\dagger \tilde{a} \tag{45}$$

The equation of motion for the operator $\tilde{a}$ may then be determined from the quantum Langevin equation

$$\dot{\tilde{a}}(t) = \frac{1}{i\hbar} \left[\tilde{a}(t), \tilde{H}_{\text{QNL}} \right] - \gamma(t)\tilde{a}(t) + \sqrt{2\gamma_{\text{in}}(t)}\tilde{a}_{\text{in}}(t) + \sqrt{2\gamma_l}\delta\tilde{a}_l(t) \tag{46}$$

$$= i \left(g_{\text{sig}} + g^{\text{self}}_{\text{therm}} + \Delta \right) \tilde{a}(t) - \gamma(t)\tilde{a}(t) + \sqrt{2\gamma_{\text{in}}(t)}\tilde{a}_{\text{in}}(t) + \sqrt{2\gamma_l}\delta\tilde{a}_l(t) \tag{47}$$

$$= \left[-\gamma(t) + i \left(g_{\text{sig}} + g^{\text{self}}_{\text{therm}} + \Delta \right) \right] \tilde{a}(t) + \sqrt{2\gamma_{\text{in}}(t)}\tilde{a}_{\text{in}}(t) + \sqrt{2\gamma_l}\delta\tilde{a}_l(t), \tag{48}$$

where we have used the Boson commutation relation $[\tilde{a}, \tilde{a}^\dagger] = 1$, $\gamma(t) = \gamma_{\text{in}}(t) + \gamma_l(t)$ is the total decay rate of the optical resonator, $\gamma_{\text{in}}(t)$ is the input coupling rate with explicit time dependence included to allow noise in this parameter to be included in the model, $\gamma_{\text{in}}(t)$ is the loss rate of the resonator, and $\tilde{a}_{\text{in}}$ and $\delta\tilde{a}_l$ are respectively the bright field entering the resonator through in the input coupler and vacuum noise entering through loss, with $\tilde{n} = \tilde{a}^\dagger_{\text{in}}\tilde{a}_{\text{in}}$ being the incident optical intensity in units of photons per second.

Input noise terms

Each of the annihilation operators representing input fields may be broken down into amplitude $\tilde{X}$ and phase $\tilde{Y}$ quadratures as

$$\tilde{a} = \frac{\tilde{X} + i\tilde{Y}}{2}. \tag{49}$$

The Boson commutation relation, results in a commutation relation between $\tilde{X}(\omega)$ and $\tilde{Y}(\omega)$, $[\tilde{X}(\omega), \tilde{Y}(\omega)] = 2i$, such that their is an uncertainty principle relating the two quadratures $\Delta\tilde{X}\Delta\tilde{Y} \geq 1$. It is this uncertainty principle which, from the perspective

of quantum mechanics, results in the shot noise limit to sensing. If the input laser is coherent at frequency ω, $\langle \delta\tilde{X}_{\text{in}}(\omega)^2\rangle = \langle \delta\tilde{Y}_{\text{in}}(\omega)^2\rangle = 1$. However, it is always the case at low frequencies of interest to biosensing experiments, that classical noise sources enter, both on the amplitude of the light and on the phase. The noise on these input fields can then be broken into a classical component and a quantum component with unity variance, eg. $\delta\tilde{X}_{\text{in}}(\omega) = \delta\tilde{X}_q(\omega) + \delta\tilde{X}_c(\omega)$, where $\langle \delta X_q^2\rangle = 1$. Taking the variance of each noise term, and using the fact that the quantum and classical noise is uncorrelated, we find

$$\left\langle \delta\tilde{X}_{\text{in}}(\omega)^2\right\rangle = \left\langle \delta\tilde{X}_q(\omega)^2\right\rangle + \left\langle \delta\tilde{X}_c(\omega)^2\right\rangle = 1 + \left\langle \delta\tilde{X}_c(\omega)^2\right\rangle \tag{50}$$

$$\left\langle \delta\tilde{Y}_{\text{in}}(\omega)^2\right\rangle = \left\langle \delta\tilde{Y}_q(\omega)^2\right\rangle + \left\langle \delta\tilde{Y}_c(\omega)^2\right\rangle = 1 + \left\langle \delta\tilde{Y}_c(\omega)^2\right\rangle \tag{51}$$

The classical phase noise can be conveniently reexpressed in terms of absolute phase noise, i.e. $\langle \delta\tilde{Y}_c(\omega)^2\rangle = V_\zeta(\omega)\alpha_{\text{in}}^2$, where $V_\zeta(\omega)$ is the variance of classical phase noise, scaled by the incident optical intensity α_{in}^2, since the absolute displacement in optical phase space is amplified by the coherent amplitude of the field. Similarly, the classical amplitude noise may be reexpressed as a relative amplitude noise $\langle \delta\tilde{X}_c(\omega)^2\rangle = V_{\text{RIN}}(\omega)\alpha_{\text{in}}^2$. It is natural to do this because the variance of classical noise in both amplitude and phase scales linearly with intensity, whilst, due to the injection of vacuum noise when the laser is attenuated for any reason, the quantum noise variance remains constant. $V_\zeta(\omega)$ and $V_{\text{RIN}}(\omega)$ are therefore intensity independent noise parameters. We then have

$$\left\langle \delta\tilde{X}_{\text{in}}(\omega)^2\right\rangle = 1 + V_{\text{RIN}}(\omega)\alpha_{\text{in}}^2 \tag{52}$$

$$\left\langle \delta\tilde{Y}_{\text{in}}(\omega)^2\right\rangle = 1 + V_\zeta(\omega)\alpha_{\text{in}}^2. \tag{53}$$

Any fluctuation in optical path length, such as these due to thermal fluctuations and mechanical vibrations, will introduce phase (or equivalently frequency) noise into the sensing apparatus. By contrast, the introduction of amplitude noise requires fluctuations in optical attenuation which are much less common. Furthermore, optical intensity fluctuations are directly measured with a photodiode, and can therefore by compensated for in measured data, or suppressed through fed back/forward as performed in common "noise eater" arrangements. Consequently, phase and frequency noise tend to be both larger in magnitude and more difficult to suppress in optical sensors [3]

Solving the mean field

The first step to solving the problem is to calculate the mean fields in the resonator and leaving it back through the input coupler. We expand each of the time varying terms in Eq. (48) into their mean values and noise fluctuations ($\tilde{a} = \langle\tilde{a}\rangle + \delta\tilde{a} = \alpha + \delta\tilde{a}$, $\gamma = \langle\gamma\rangle + \delta\gamma = \bar{\gamma} + \delta\gamma$), take the expectation value, and linearize by neglecting noise product terms to get

$$\dot{\alpha}(t) = \left(-\bar{\gamma} + i(g_{\text{sig}} + \Delta)\right)\alpha(t) + \sqrt{2\bar{\gamma}_{\text{in}}}\alpha_{\text{in}}(t), \tag{54}$$

where, since $g_{\text{therm}}^{\text{self}}$ is zero mean thermorefractive noise $\langle g_{\text{therm}}^{\text{self}}\rangle = 0$. Choosing the input field mean amplitude to be stationary and taking the steady state ($\alpha_{\text{in}}(t) = \alpha_{\text{in}}$, $\dot{\alpha}(t) = 0$), we get

$$0 = \left(-\bar{\gamma} + i(g_{\text{sig}} + \Delta)\right)\alpha + \sqrt{2\bar{\gamma}_{\text{in}}}\alpha_{\text{in}} \tag{55}$$

where henceforth α is implicitly taken to be the steady state intracavity intensity. So that

$$\alpha = \frac{\sqrt{2\bar{\gamma}_{\text{in}}}\alpha_{\text{in}}}{\bar{\gamma} - i(g_{\text{sig}} + \Delta)} \tag{56}$$

The limit relevant to high precision sensing experiments is the limit where both the frequency shift due to the nanoparticle g_{sig} and the cavity detuning are small compared to the cavity linewidth $\bar{\gamma}$. Therefore, it is reasonable to take the limit $\{g_{\text{sig}}, \Delta\} \ll \bar{\gamma}$. In this case, it is straightforward to show that Eq. (56) may be well approximated as

$$\alpha = \frac{\sqrt{2\bar{\gamma}_{\text{in}}}}{\bar{\gamma}^2}\left(\bar{\gamma} + i(g_{\text{sig}} + \Delta)\right)\alpha_{\text{in}} \tag{57}$$

The mean amplitude of the output field may be calculated using the input-output formalism with

$$\tilde{a}_{\text{out}} = \tilde{a}_{\text{in}} - \sqrt{2\gamma_{\text{in}}(t)}\tilde{a}. \tag{58}$$

Since $\gamma_{\rm in}$ varies with time but the amplitude of variation is small compared with the coupling rate itself, the square-root can be expanded as $\sqrt{2\gamma_{\rm in}(t)} \approx \sqrt{2\bar{\gamma}_{\rm in}} + \delta\gamma_{\rm in}(t)/\sqrt{2\bar{\gamma}_{\rm in}}$, so that

$$\tilde{a}_{\rm out} = \tilde{a}_{\rm in} - \left[\sqrt{2\bar{\gamma}_{\rm in}} + \frac{\delta\gamma_{\rm in}}{\sqrt{2\bar{\gamma}_{\rm in}}}\right]\tilde{a}. \tag{59}$$

This results in mean and fluctuation input output relations

$$\alpha_{\rm out} = \alpha_{\rm in} - \sqrt{2\bar{\gamma}_{\rm in}}\alpha \tag{60}$$
$$\delta\tilde{a}_{\rm out} = \delta\tilde{a}_{\rm in} - \sqrt{2\bar{\gamma}_{\rm in}}\delta\tilde{a} - \frac{\alpha}{\sqrt{2\bar{\gamma}_{\rm in}}}\delta\gamma_{\rm in}. \tag{61}$$

Using Eqs. (57) and (60) we then find

$$\alpha_{\rm out} = \alpha_{\rm in} - \frac{2\bar{\gamma}_{\rm in}}{\bar{\gamma}^2}\left(\bar{\gamma} + i(g_{\rm sig} + \Delta)\right)\alpha_{\rm in} \tag{62}$$
$$= \left[1 - 2\eta\left(1 + i(g'_{\rm sig} + \Delta')\right)\right]\alpha_{\rm in} \tag{63}$$

where it will be convenient throughout to normalize rates in terms of the mean cavity decay rate $\bar{\gamma}$. In these cases, the normalization will generally be denoted with a $'$. Here, for example $g'_{\rm sig} = g_{\rm sig}/\bar{\gamma}$ and ${g_{\rm therm}^{\rm self}}' = g_{\rm therm}^{\rm self}/\bar{\gamma}$. $\eta = \bar{\gamma}_{\rm in}/\bar{\gamma}$ is the escape efficiency of the cavity, i.e. the probability that a photon in the cavity will leave through the input/output coupler. Since we wish to measure the magnitude of $g_{\rm sig}$ and $g_{\rm sig} \ll \bar{\gamma}$, it is clear from Eq. (63) that we wish to measure a phase rotation on the output field of the resonator ($g_{\rm sig}$ displaces the amplitude of the field a small amount in a direction in phase space orthogonal to the mean amplitude).

Solving for the fluctuating noise

To solve for the steady-state fluctuations on both intracavity and output fields we make the substitutions $\tilde{a} = \alpha + \delta\tilde{a}$ for each annihilation operator in Eq. 48, substitute $\gamma(t) = \bar{\gamma} + \delta\gamma(t)$, and substitute $\sqrt{2\gamma_{\rm in}(t)} = \sqrt{2\bar{\gamma}_{\rm in}} + \delta\gamma_{\rm in}(t)/\sqrt{2\bar{\gamma}_{\rm in}}$. Using Eq. (57) the mean terms cancel, neglecting second order fluctuating terms we find

$$\delta\dot{\tilde{a}}(t) = i(g_{\rm sig} + \Delta)\delta\tilde{a}(t) - \bar{\gamma}\delta\tilde{a}(t) - \alpha\delta\gamma_{\rm in}(t) + i\alpha g_{\rm therm}^{\rm self}(t) + \frac{\alpha_{\rm in}}{\sqrt{2\bar{\gamma}_{\rm in}}}\delta\gamma_{\rm in}(t) + \sqrt{2\bar{\gamma}_{\rm in}}\delta\tilde{a}_{\rm in}(t) + \sqrt{2\gamma_l}\delta\tilde{a}_l(t) \tag{64}$$

This can easily be solved in the frequency domain by taking the Fourier transform, which yields

$$\delta\tilde{a}(\omega) = \left[\frac{1}{\bar{\gamma} + i(\omega - g_{\rm sig} - \Delta)}\right]\left\{\frac{\alpha_{\rm in}}{\sqrt{2\bar{\gamma}_{\rm in}}}\left[1 - \frac{2\bar{\gamma}_{\rm in}}{\bar{\gamma} - i(g_{\rm sig} + \Delta)}\right]\delta\gamma_{\rm in}(\omega) + i\alpha g_{\rm therm}^{\rm self}(\omega) + \sqrt{2\bar{\gamma}_{\rm in}}\delta\tilde{a}_{\rm in}(\omega) + \sqrt{2\gamma_l}\delta\tilde{a}_l(\omega)\right\} \tag{65}$$
$$= \left[\frac{\bar{\gamma} - i(\omega - g_{\rm sig} - \Delta)}{\bar{\gamma}^2}\right]\left\{\frac{\alpha_{\rm in}}{\sqrt{2\bar{\gamma}_{\rm in}}}\left[1 - \frac{2\bar{\gamma}_{\rm in}}{\bar{\gamma} - i(g_{\rm sig} + \Delta)}\right]\delta\gamma_{\rm in} + i\alpha_{\rm in}\frac{\sqrt{2\bar{\gamma}_{\rm in}}}{\bar{\gamma}}g_{\rm therm}^{\rm self} + \sqrt{2\bar{\gamma}_{\rm in}}\delta\tilde{a}_{\rm in} + \sqrt{2\gamma_l}\delta\tilde{a}_l\right\} \tag{66}$$
$$= \left[\frac{\bar{\gamma} - i(\omega - g_{\rm sig} - \Delta)}{\bar{\gamma}^2}\right]\left\{\frac{\alpha_{\rm in}}{\sqrt{2\bar{\gamma}_{\rm in}}}\left[1 - \frac{2\bar{\gamma}_{\rm in}(\bar{\gamma} + i(g_{\rm sig} + \Delta))}{\bar{\gamma}^2}\right]\delta\gamma_{\rm in} + i\alpha_{\rm in}\frac{\sqrt{2\bar{\gamma}_{\rm in}}}{\bar{\gamma}}g_{\rm therm}^{\rm self} + \sqrt{2\bar{\gamma}_{\rm in}}\delta\tilde{a}_{\rm in} + \sqrt{2\gamma_l}\delta\tilde{a}_l\right\} \tag{67}$$
$$= \left[\frac{1 - i(\omega' - g'_{\rm sig} - \Delta')}{\bar{\gamma}}\right]\left\{\frac{\alpha_{\rm in}}{\sqrt{2\bar{\gamma}_{\rm in}}}\left[1 - 2\eta - 2i\eta(g'_{\rm sig} + \Delta')\right]\delta\gamma_{\rm in} + i\alpha_{\rm in}\sqrt{2\bar{\gamma}_{\rm in}}{g_{\rm therm}^{\rm self}}' + \sqrt{2\bar{\gamma}_{\rm in}}\delta\tilde{a}_{\rm in} + \sqrt{2\gamma_l}\delta\tilde{a}_l\right\}, \tag{68}$$

where we have substituted in for α from Eq. (57), neglected the term involving the product $(g_{\rm sig} + \Delta)g_{\rm therm}^{\rm self}$ since this is small compared with $\bar{\gamma}^2$, and taken $\omega \ll \bar{\gamma}$. For compactness we've omitted the explicit frequency dependence ω of the fluctuations here and henceforth.

The output field fluctuations can then be found using Eq. (61)

$$\delta\tilde{a}_{\rm out}(\omega) = \delta\tilde{a}_{\rm in} - \left[\frac{1-i(\omega'-g'_{\rm sig}-\Delta')}{\bar{\gamma}}\right]\left\{\alpha_{\rm in}\left[1-2\eta-2i\eta(g'_{\rm sig}+\Delta')\right]\delta\gamma_{\rm in}+2i\bar{\gamma}_{\rm in}\alpha_{\rm in}g^{\rm self}_{\rm therm}{}'+2\bar{\gamma}_{\rm in}\delta\tilde{a}_{\rm in}+2\sqrt{\gamma_{\rm in}\gamma_l}\delta\tilde{a}_l\right\} \quad (69)$$

$$-\frac{\alpha}{\sqrt{2\bar{\gamma}_{\rm in}}}\delta\gamma_{\rm in} \quad (70)$$

$$= \delta\tilde{a}_{\rm in} - \left[\frac{1-i(\omega'-g'_{\rm sig}-\Delta')}{\bar{\gamma}}\right]\left\{\alpha_{\rm in}\left[1-2\eta-2i\eta(g'_{\rm sig}+\Delta')\right]\delta\gamma_{\rm in}+2i\bar{\gamma}_{\rm in}\alpha_{\rm in}g^{\rm self}_{\rm therm}{}'+2\bar{\gamma}_{\rm in}\delta\tilde{a}_{\rm in}+2\sqrt{\gamma_{\rm in}\gamma_l}\delta\tilde{a}_l\right\} \quad (71)$$

$$-\frac{1}{\bar{\gamma}}\left(1+i(g'_{\rm sig}+\Delta')\right)\alpha_{\rm in}\delta\gamma_{\rm in} \quad (72)$$

$$= \delta\tilde{a}_{\rm in} - \left(1-i(\omega'-g'_{\rm sig}-\Delta')\right)\left\{\alpha_{\rm in}\left[1-2\eta-2i\eta(g'_{\rm sig}+\Delta')\right]\delta\gamma'_{\rm in}+2i\eta\alpha_{\rm in}g^{\rm self}_{\rm therm}{}'+2\eta\delta\tilde{a}_{\rm in}+2\sqrt{\eta(1-\eta)}\delta\tilde{a}_l\right\} \quad (73)$$

$$-\left(1+i(g'_{\rm sig}+\Delta')\right)\alpha_{\rm in}\delta\gamma'_{\rm in} \quad (74)$$

$$= \left[1-2\eta\left(1-i(\omega'-g'_{\rm sig}-\Delta')\right)\right]\delta\tilde{a}_{\rm in}-2\sqrt{\eta(1-\eta)}\left(1-i(\omega'-g'_{\rm sig}-\Delta')\right)\delta a_l \quad (75)$$

$$-\alpha_{\rm in}\left[1+i(g'_{\rm sig}+\Delta')+\left(1-i(\omega'-g'_{\rm sig}-\Delta')\right)\left(1-2\eta-2i\eta(g'_{\rm sig}+\Delta')\right)\right]\delta\gamma'_{\rm in} \quad (76)$$

$$-2i\eta\alpha_{\rm in}\left(1-i(\omega'-g'_{\rm sig}-\Delta')\right)g^{\rm self}_{\rm therm}{}' \quad (77)$$

The output field annihilation operator is given by $\tilde{a}_{\rm out} = \alpha_{\rm out} + \delta\tilde{a}_{\rm out}$. As can be seen from Eq. (63), the signal $g'_{\rm sig}$ is contained entirely within the imaginary part of $\alpha_{\rm out}$. It can be shown from Eq. (77) that the noise variance is independent of the measured phase. Consequently, the optimal approach to extracting a signal from $\tilde{a}_{\rm out}$ is to measure the phase quadrature $\tilde{Y}_{\rm out}(\omega) = i\left[\tilde{a}^\dagger_{\rm out}(-\omega)-\tilde{a}_{\rm out}(\omega)\right]$. From Eq. (63) we have

$$\left\langle\tilde{Y}_{\rm out}\right\rangle = -4\eta\alpha_{\rm in}\left(g'_{\rm sig}+\Delta'\right) \quad (78)$$

Thus, an estimate of $g'_{\rm sig}$ may be obtained from a measurement of $\tilde{Y}_{\rm out}$ as

$$g^{\rm est\,\prime}_{\rm sig} = -\frac{\tilde{Y}_{\rm out}}{4\eta\alpha_{\rm in}}-\Delta' = -\frac{\left\langle\tilde{Y}_{\rm out}\right\rangle}{4\eta\alpha_{\rm in}}-\Delta'-\frac{\delta\tilde{Y}_{\rm out}}{4\eta\alpha_{\rm in}} \quad (79)$$

which in the limit that no noise was present in the measurement, would exactly retrieve $g'_{\rm sig}$. It can be seen from this equation that the variance, or uncertainty, of the estimate is given by

$$\left\langle\left|\delta g^{\rm est\,\prime}_{\rm sig}\right|^2\right\rangle_{\rm QNL} = \left\langle\left|g^{\rm est\,\prime}_{\rm sig}\right|^2\right\rangle-\left|\left\langle g^{\rm est\,\prime}_{\rm sig}\right\rangle\right|^2 = \frac{\left\langle\left|\delta\tilde{Y}_{\rm out}\right|^2\right\rangle}{16\eta^2\alpha^2_{\rm in}} \quad (80)$$

From Eq. (77) we have

$$\delta\tilde{Y}_{\rm out}(\omega) = -2\eta(g'_{\rm sig}+\Delta')\delta\tilde{X}_{\rm in}+\left[1-2\eta\left(1-i\omega'\right)\right]\delta\tilde{Y}_{\rm in}-2\sqrt{\eta(1-\eta)}\left[(g'_{\rm sig}+\Delta')\delta X_l+\left(1-i\omega'\right)\delta Y_l\right] \quad (81)$$

$$-4\alpha_{\rm in}\left(g'_{\rm sig}+\Delta'\right)\left[1-2\eta-i\eta\omega'\right]\delta\gamma'_{\rm in}-4\eta\alpha_{\rm in}\left(1-i\omega'\right)g^{\rm self}_{\rm therm}{}' \quad (82)$$

where we have used the relations $\delta\gamma'_{\rm in}(-\omega)^\dagger = \delta\gamma'_{\rm in}(\omega)$ and $g^{\rm self}_{\rm therm}{}'(-\omega)^\dagger = g^{\rm self}_{\rm therm}{}'(\omega)$ which arise since both the input coupling rate and thermal fluctuations are real parameters in the time domain; and in general $\tilde{X}_{\rm out}(\omega) = \tilde{a}^\dagger(-\omega)+\tilde{a}(\omega)$, and $\tilde{Y}(\omega) = i\left[\tilde{a}^\dagger(-\omega)-\tilde{a}(\omega)\right]$. Given that each of the fluctuating terms in this expression are uncorrelated, the output phase quadrature variance is

$$\left\langle\left|\delta\tilde{Y}_{\rm out}(\omega)\right|^2\right\rangle = 4\eta^2(g'_{\rm sig}+\Delta')^2\left\langle\delta\tilde{X}^2_{\rm in}\right\rangle+\left[(1-2\eta)^2+4\eta^2\omega'^2\right]\left\langle\delta\tilde{Y}^2_{\rm in}\right\rangle+4\eta(1-\eta)\left[(g'_{\rm sig}+\Delta')^2\left\langle\delta X^2_l\right\rangle+\left(1+\omega'^2\right)\left\langle\delta Y^2_l\right\rangle\right] \quad (83)$$

$$+16\alpha^2_{\rm in}\left(g'_{\rm sig}+\Delta'\right)^2\left[(1-2\eta)^2+\eta^2\omega'^2\right]\left\langle\delta\gamma'^2_{\rm in}\right\rangle+16\eta^2\alpha^2_{\rm in}\left(1+\omega'^2\right)\left\langle g^{\rm self}_{\rm therm}{}'^2\right\rangle \quad (84)$$

The laser input variances can be broken into a quantum part and a classical part, as described by Eqs. (52) and (53); whilst the fields entering through the sensor loss channel are vacuum and therefore quantum noise limited ($\langle\delta X^2_l\rangle = \langle\delta Y^2_l\rangle = 1$). Using

these relations we find

$$\left\langle\left|\delta\tilde{Y}_{\rm out}(\omega)\right|^2\right\rangle = 4\eta^2(g'_{\rm sig}+\Delta')^2\left(1+V_{\rm RIN}\alpha_{\rm in}^2\right)+\left[(1-2\eta)^2+4\eta^2\omega'^2\right]\left(1+V_\zeta\alpha_{\rm in}^2\right) \quad (85)$$

$$+4\eta(1-\eta)\left[(g'_{\rm sig}+\Delta')^2+\left(1+\omega'^2\right)\right] \quad (86)$$

$$+16\alpha_{\rm in}^2\left(g'_{\rm sig}+\Delta'\right)^2\left[(1-2\eta)^2+\eta^2\omega'^2\right]\left\langle\delta\gamma'_{\rm in}{}^2\right\rangle+16\eta^2\alpha_{\rm in}^2\left(1+\omega'^2\right)\left\langle g_{\rm therm}^{\rm self}{}'^2\right\rangle \quad (87)$$

$$= 4\eta^2(g'_{\rm sig}+\Delta')^2V_{\rm RIN}\alpha_{\rm in}^2+\left[(1-2\eta)^2+4\eta^2\omega'^2\right]V_\zeta\alpha_{\rm in}^2 \quad (88)$$

$$+16\alpha_{\rm in}^2\left(g'_{\rm sig}+\Delta'\right)^2\left[(1-2\eta)^2+\eta^2\omega'^2\right]\left\langle\delta\gamma'_{\rm in}{}^2\right\rangle+16\eta^2\alpha_{\rm in}^2\left(1+\omega'^2\right)\left\langle g_{\rm therm}^{\rm self}{}'^2\right\rangle \quad (89)$$

$$+1+4\eta\left[(g'_{\rm sig}+\Delta')^2+\omega'^2\right] \quad (90)$$

Taking the reasonable limit that $\{g_{\rm sig}', \Delta', \omega'\} \ll 1$, and that the presence of the particle to be sensed does not effect the noise (i.e. neglecting all noise terms multiplied by $g_{\rm sig}'$), we have

$$\left\langle\left|\delta\tilde{Y}_{\rm out}(\omega)\right|^2\right\rangle = 4\eta^2\alpha_{\rm in}^2\Delta'^2V_{\rm RIN}+\alpha_{\rm in}^2(1-2\eta)^2V_\zeta+16\alpha_{\rm in}^2\Delta'^2(1-2\eta)^2\left\langle\delta\gamma'_{\rm in}{}^2\right\rangle+16\eta^2\alpha_{\rm in}^2\left\langle g_{\rm therm}^{\rm self}{}'^2\right\rangle+1. \quad (91)$$

This expression shows the effect of each of the four sources of noise on variance of the output field. Notice that since $\Delta' \ll 1$, the relative intensity noise $V_{\rm RIN}$ is greatly suppressed compared to the laser phase noise V_ζ in this measurement.

The uncertainty in our estimate of $g'_{\rm sig}$ is then finally

$$\left\langle\left|\delta g_{\rm sig}^{\rm est}{}'\right|^2\right\rangle_{\rm QNL} = \left(\frac{\Delta'}{2}\right)^2V_{\rm RIN}+\left(\frac{1-2\eta}{4\eta}\right)^2V_\zeta+\Delta'^2\left(\frac{1-2\eta}{\eta}\right)^2\left\langle\delta\gamma'_{\rm in}{}^2\right\rangle+\left\langle g_{\rm therm}^{\rm self}{}'^2\right\rangle+\frac{1}{16\eta^2\alpha_{\rm in}^2} \quad (92)$$

$$= \left(\frac{\Delta'}{2}\right)^2V_{\rm RIN}+\left(\frac{1-2\eta}{4\eta}\right)^2V_\zeta+\Delta'^2\left(\frac{1-2\eta}{\eta}\right)^2V_\gamma+V_{\rm therm}+\frac{1}{16\eta^2\alpha_{\rm in}^2}, \quad (93)$$

where for compactness we have defined $\left\langle\delta\gamma'_{\rm in}{}^2\right\rangle = V_\gamma$ and $\left\langle g_{\rm therm}^{\rm self}{}'^2\right\rangle = V_{\rm therm}$. From this expression one sees that, as expected, only the contribution of shot noise towards the uncertainty (the final term) depends on laser power. Of the other terms, it should be expected that laser phase noise dominates, since this is typically large in the frequency range of interest to biosensing experiments and is not suppressed by the measurement technique in the same way that intensity noise is.

BACKSCATTER MEASUREMENT

To perform a similar calculation to that performed above for backscatter measurement, we return to the full Hamiltonian in Eq. (24). The equations of motion for the two intracavity annihilation operators are then

$$\dot{\hat{a}}_A = \frac{1}{i\hbar}\left[\hat{a}_A, H\right]-\gamma(t)\hat{a}_A+\sqrt{2\gamma_{\rm in}(t)}\hat{a}_{A,{\rm in}}+\sqrt{2\gamma_l}\delta\hat{a}_{A,l} \quad (94)$$

$$= i\left(g_{\rm sig}+g_{\rm therm}^{\rm self}+\Delta\right)\hat{a}_A+i\left(g_{\rm sig}e^{2im\phi_{\rm sig}}+g_0e^{2im\phi_0}+g_{\rm therm}^{\rm cross}\right)\hat{a}_C-\gamma(t)\hat{a}_A+\sqrt{2\gamma_{\rm in}(t)}\hat{a}_{A,{\rm in}}+\sqrt{2\gamma_l}\delta\hat{a}_{A,l} \quad (95)$$

$$\dot{\hat{a}}_C = \frac{1}{i\hbar}\left[\hat{a}_C, H\right]-\gamma(t)\hat{a}_C+\sqrt{2\gamma_{\rm in}(t)}\delta\hat{a}_{C,{\rm in}}+\sqrt{2\gamma_l}\delta\hat{a}_{C,l} \quad (96)$$

$$= i\left(g_{\rm sig}+g_{\rm therm}^{\rm self}+\Delta\right)\hat{a}_C+i\left(g_{\rm sig}e^{-2im\phi_{\rm sig}}+g_0e^{-2im\phi_0}+g_{\rm therm}^{\rm cross\,*}\right)\hat{a}_A-\gamma(t)\hat{a}_C+\sqrt{2\gamma_{\rm in}(t)}\delta\hat{a}_{C,{\rm in}}+\sqrt{2\gamma_l}\delta\hat{a}_{C,l} \quad (97)$$

Solving the mean fields

Similar to the above, we can find the steady state mean fields by taking the expectation values of Eqs. (95) and (97), setting the time derivative to zero, and solving. From Eq. (95) we find

$$\alpha_A\left[\bar{\gamma}-i\left(g_{\rm sig}+\Delta\right)\right] = i\left(g_{\rm sig}e^{2im\phi_{\rm sig}}+g_0e^{2im\phi_0}\right)\alpha_C+\sqrt{2\bar{\gamma}_{\rm in}}\alpha_{A,{\rm in}} \quad (98)$$

Rearranging we find

$$\alpha_A = \frac{i\left(g_{\rm sig}e^{2im\phi_{\rm sig}}+g_0e^{2im\phi_0}\right)\alpha_C+\sqrt{2\bar{\gamma}_{\rm in}}\alpha_{A,{\rm in}}}{\bar{\gamma}-i\left(g_{\rm sig}+\Delta\right)}. \quad (99)$$

Similarly for α_C we have

$$\alpha_C = \frac{i\left(g_{\rm sig}e^{-2im\phi_{\rm sig}} + g_0 e^{-2im\phi_0}\right)}{\bar{\gamma} - i\left(g_{\rm sig} + \Delta\right)}\alpha_A, \tag{100}$$

where here, we consider a scenario where only the anti-clockwise mode is directly pumped, and therefore $\alpha_{C,\rm in} = 0$. It is then straightforward to solve for α_C as

$$\alpha_C = \left[\frac{i\left(g_{\rm sig}e^{-2im\phi_{\rm sig}} + g_0 e^{-2im\phi_0}\right)}{\bar{\gamma} - i\left(g_{\rm sig} + \Delta\right)}\right]\left[\frac{i\left(g_{\rm sig}e^{2im\phi_{\rm sig}} + g_0 e^{2im\phi_0}\right)\alpha_C + \sqrt{2\bar{\gamma}_{\rm in}}\alpha_{A,\rm in}}{\bar{\gamma} - i\left(g_{\rm sig} + \Delta\right)}\right], \tag{101}$$

$$\left[1 + \frac{\left|g_{\rm sig}e^{2im\phi_{\rm sig}} + g_0 e^{2im\phi_0}\right|^2}{\left(\bar{\gamma} - i\left(g_{\rm sig} + \Delta\right)\right)^2}\right]\alpha_C = \left[\frac{i\sqrt{2\bar{\gamma}_{\rm in}}\left(g_{\rm sig}e^{-2im\phi_{\rm sig}} + g_0 e^{-2im\phi_0}\right)}{\left(\bar{\gamma} - i\left(g_{\rm sig} + \Delta\right)\right)^2}\right]\alpha_{A,\rm in} \tag{102}$$

$$\alpha_C = \left[\frac{i\sqrt{2\bar{\gamma}_{\rm in}}\left(g_{\rm sig}e^{-2im\phi_{\rm sig}} + g_0 e^{-2im\phi_0}\right)}{\left(\bar{\gamma} - i\left(g_{\rm sig} + \Delta\right)\right)^2 + \left|g_{\rm sig}e^{2im\phi_{\rm sig}} + g_0 e^{2im\phi_0}\right|^2}\right]\alpha_{A,\rm in} \tag{103}$$

$$= \left[\frac{i\sqrt{2\bar{\gamma}_{\rm in}}\left(g_{\rm sig}e^{-2im\phi_{\rm sig}} + g_0 e^{-2im\phi_0}\right)}{(\bar{\gamma} - i\Delta)^2}\right]\alpha_{A,\rm in} \tag{104}$$

$$= i\frac{\sqrt{2\bar{\gamma}_{\rm in}}}{(\bar{\gamma} - i\Delta)^2}\left(g_{\rm sig}e^{-2im\phi_{\rm sig}} + g_0 e^{-2im\phi_0}\right)\alpha_{A,\rm in} \tag{105}$$

$$\tag{106}$$

where we've used the assumption that $\{g_{\rm sig}, g_0\} \ll \bar{\gamma}$. We can then find the intracavity field amplitude in mind A

$$\alpha_A = \left[\frac{-\sqrt{2\bar{\gamma}_{\rm in}}\left|g_{\rm sig}e^{2im\phi_{\rm sig}} + g_0 e^{2im\phi_0}\right|^2 / (\bar{\gamma} - i\Delta)^2 + \sqrt{2\bar{\gamma}_{\rm in}}}{\bar{\gamma} - i\left(g_{\rm sig} + \Delta\right)}\right]\alpha_{A,\rm in} \tag{107}$$

$$= \sqrt{2\bar{\gamma}_{\rm in}}\left[\frac{(\bar{\gamma} - i\Delta)^2 - \left|g_{\rm sig}e^{2im\phi_{\rm sig}} + g_0 e^{2im\phi_0}\right|^2}{\left(\bar{\gamma} - i\left(g_{\rm sig} + \Delta\right)\right)(\bar{\gamma} - i\Delta)^2}\right]\alpha_{A,\rm in} \tag{108}$$

$$\approx \frac{\sqrt{2\bar{\gamma}_{\rm in}}}{(\bar{\gamma} - i\Delta)}\left[1 - \frac{g_0^2}{(\bar{\gamma} - i\Delta)^2}\right]\alpha_{A,\rm in}, \tag{109}$$

Where we have, as usual, assumed that $g_{\rm sig} \ll \{g_0, \bar{\gamma}\}$. Furthermore, since $g_0 \ll \bar{\gamma}$ we immediately see that $\alpha_A \gg \alpha_C$. One might, therefore think that a higher signal-to-noise could be obtained by detecting α_A that α_C. However, this is not this case. In fact, when the incident field is on resonance with the cavity resonance any signal arising from the field output from mode A is second order in $g_{\rm sig}$, whereas that arising from the field output from mode C is first order.

Since $\alpha_{C,\rm out} = \alpha_{C,\rm in} - \sqrt{2\bar{\gamma}_{\rm in}}\alpha_C$, and $\alpha_{C,\rm in} = 0$ we have

$$\alpha_{C,\rm out} = -\frac{2i\eta}{(1 - i\Delta')^2}\left(g'_{\rm sig}e^{-2im\phi_{\rm sig}} + g'_0 e^{-2im\phi_0}\right)\alpha_{A,\rm in} \tag{110}$$

Solving for the fluctuating noise

Taking the fluctuating parts of Eqs. (95) and (97) we have

$$\delta\dot{\hat{a}}_A = -\left[\bar{\gamma} - i\left(g_{\rm sig} + \Delta\right)\right]\delta\hat{a}_A + i\left(g_{\rm sig}e^{2im\phi_{\rm sig}} + g_0 e^{2im\phi_0}\right)\delta\hat{a}_C + i\alpha_A g_{\rm therm}^{\rm self} + i\alpha_C g_{\rm therm}^{\rm cross} + \left[\frac{\alpha_{A,\rm in}}{\sqrt{2\bar{\gamma}_{\rm in}}} - \alpha_A\right]\delta\gamma_{\rm in} + \sqrt{2\bar{\gamma}_{\rm in}}\delta a_{A,\rm in} + \sqrt{2\gamma_l}\delta\hat{a}_{A,l}$$

$$\delta\dot{\hat{a}}_C = -\left[\bar{\gamma} - i\left(g_{\rm sig} + \Delta\right)\right]\delta\hat{a}_C + i\left(g_{\rm sig}e^{-2im\phi_{\rm sig}} + g_0 e^{-2im\phi_0}\right)\delta\hat{a}_A + i\alpha_C g_{\rm therm}^{\rm self} + i\alpha_A g_{\rm therm}^{\rm cross\,*} - \alpha_C\delta\gamma_{\rm in} + \sqrt{2\bar{\gamma}_{\rm in}}\delta a_{C,\rm in} + \sqrt{2\gamma_l}\delta\hat{a}_{C,l}$$

Taking the Fourier transform and rearranging we find

$$\delta\tilde{a}_A = \frac{1}{\bar{\gamma} + i\left(\omega - g_{\rm sig} - \Delta\right)}\left\{i\left(g_{\rm sig}e^{2im\phi_{\rm sig}} + g_0 e^{2im\phi_0}\right)\delta\tilde{a}_C + i\alpha_A g_{\rm therm}^{\rm self} + i\alpha_C g_{\rm therm}^{\rm cross} + \left[\frac{\alpha_{A,\rm in}}{\sqrt{2\bar{\gamma}_{\rm in}}} - \alpha_A\right]\delta\gamma_{\rm in} + \sqrt{2\bar{\gamma}_{\rm in}}\delta\tilde{a}_{A,\rm in} + \sqrt{2\gamma_l}\delta\tilde{a}_{A,l}\right\}$$

$$\delta\tilde{a}_C = \frac{1}{\bar{\gamma} + i\left(\omega - g_{\rm sig} - \Delta\right)}\left\{i\left(g_{\rm sig}e^{-2im\phi_{\rm sig}} + g_0 e^{-2im\phi_0}\right)\delta\tilde{a}_A + i\alpha_C g_{\rm therm}^{\rm self} + i\alpha_A g_{\rm therm}^{\rm cross\,*} - \alpha_C\delta\gamma_{\rm in} + \sqrt{2\bar{\gamma}_{\rm in}}\delta\tilde{a}_{C,\rm in} + \sqrt{2\gamma_l}\delta\tilde{a}_{C,l}\right\}$$

Substituting the first of these equations into the second, we can find an expression for $\delta\hat{a}_C$ in terms of the input noise sources.

$$\left[\bar{\gamma} + i\left(\omega - g_{\rm sig} - \Delta\right)\right]\delta\tilde{a}_C = \frac{i\left(g_{\rm sig}e^{-2im\phi_{\rm sig}} + g_0 e^{-2im\phi_0}\right)}{\bar{\gamma} + i\left(\omega - g_{\rm sig} - \Delta\right)}\left\{i\left(g_{\rm sig}e^{2im\phi_{\rm sig}} + g_0 e^{2im\phi_0}\right)\delta\tilde{a}_C + i\alpha_A g_{\rm therm}^{\rm self} + i\alpha_C g_{\rm therm}^{\rm cross} + \left[\frac{\alpha_{A,\rm in}}{\sqrt{2\bar{\gamma}_{\rm in}}} - \alpha_A\right]\delta\gamma_{\rm in}\right.$$
$$\left. + \sqrt{2\bar{\gamma}_{\rm in}}\delta\tilde{a}_{A,\rm in} + \sqrt{2\gamma_l}\delta\tilde{a}_{A,l}\right\} + i\alpha_C g_{\rm therm}^{\rm self} + i\alpha_A g_{\rm therm}^{\rm cross\,*} - \alpha_C\delta\gamma_{\rm in} + \sqrt{2\bar{\gamma}_{\rm in}}\delta\tilde{a}_{C,\rm in} + \sqrt{2\gamma_l}\delta\tilde{a}_{C,l}$$

$$\left\{\left[\bar{\gamma} + i\left(\omega - g_{\rm sig} - \Delta\right)\right]^2 + \left|g_{\rm sig}e^{2im\phi_{\rm sig}} + g_0 e^{2im\phi_0}\right|^2\right\}\delta\tilde{a}_C = \tag{111}$$
$$i\left(g_{\rm sig}e^{-2im\phi_{\rm sig}} + g_0 e^{-2im\phi_0}\right)\left\{i\alpha_A g_{\rm therm}^{\rm self} + i\alpha_C g_{\rm therm}^{\rm cross} + \left[\frac{\alpha_{A,\rm in}}{\sqrt{2\bar{\gamma}_{\rm in}}} - \alpha_A\right]\delta\gamma_{\rm in} + \sqrt{2\bar{\gamma}_{\rm in}}\delta\tilde{a}_{A,\rm in} + \sqrt{2\gamma_l}\delta\tilde{a}_{A,l}\right\}$$
$$+ \left[\bar{\gamma} + i\left(\omega - g_{\rm sig} - \Delta\right)\right]\left\{i\alpha_C g_{\rm therm}^{\rm self} + i\alpha_A g_{\rm therm}^{\rm cross\,*} - \alpha_C\delta\gamma_{\rm in} + \sqrt{2\bar{\gamma}_{\rm in}}\delta\tilde{a}_{C,\rm in} + \sqrt{2\gamma_l}\delta\tilde{a}_{C,l}\right\}$$

$$\left[\bar{\gamma} + i\left(\omega - g_{\rm sig} - \Delta\right)\right]^2\delta\tilde{a}_C \approx \left\{i\alpha_C\left[\bar{\gamma} + i\left(\omega - g_{\rm sig} - \Delta\right)\right] - \alpha_A g_0 e^{-2im\phi_0}\right\}g_{\rm therm}^{\rm self} + i\alpha_A\left[\bar{\gamma} + i\left(\omega - g_{\rm sig} - \Delta\right)\right]g_{\rm therm}^{\rm cross\,*} - \alpha_C g_0 e^{-2im\phi_0}g_{\rm therm}^{\rm cross}$$
$$-\left\{\alpha_C\left[\bar{\gamma} + i\left(\omega - g_{\rm sig} - \Delta\right)\right] - ig_0 e^{-2im\phi_0}\left[\frac{\alpha_{A,\rm in}}{\sqrt{2\bar{\gamma}_{\rm in}}} - \alpha_A\right]\right\}\delta\gamma_{\rm in} \tag{112}$$
$$+ig_0 e^{-2im\phi_0}\left(\sqrt{2\bar{\gamma}_{\rm in}}\delta\tilde{a}_{A,\rm in} + \sqrt{2\gamma_l}\delta\tilde{a}_{A,l}\right) + \left[\bar{\gamma} + i\left(\omega - g_{\rm sig} - \Delta\right)\right]\left(\sqrt{2\bar{\gamma}_{\rm in}}\delta\tilde{a}_{C,\rm in} + \sqrt{2\gamma_l}\delta\tilde{a}_{C,l}\right) \tag{113}$$

Substituting $g_{\rm therm}^{\rm cross} = R_{\rm therm}^{\rm cross} + iI_{\rm therm}^{\rm cross}$, we have

$$\left[\bar{\gamma} + i\left(\omega - g_{\rm sig} - \Delta\right)\right]^2\delta\tilde{a}_C = \left\{i\alpha_C\left[\bar{\gamma} + i\left(\omega - g_{\rm sig} - \Delta\right)\right] - \alpha_A g_0 e^{-2im\phi_0}\right\}g_{\rm therm}^{\rm self} \tag{114}$$
$$+\left\{i\alpha_A\left[\bar{\gamma} + i\left(\omega - g_{\rm sig} - \Delta\right)\right] - \alpha_C g_0 e^{-2im\phi_0}\right\}R_{\rm therm}^{\rm cross} \tag{115}$$
$$+\left\{\alpha_A\left[\bar{\gamma} + i\left(\omega - g_{\rm sig} - \Delta\right)\right] - i\alpha_C g_0 e^{-2im\phi_0}\right\}I_{\rm therm}^{\rm cross} \tag{116}$$
$$-\left\{\alpha_C\left[\bar{\gamma} + i\left(\omega - g_{\rm sig} - \Delta\right)\right] - ig_0 e^{-2im\phi_0}\left[\frac{\alpha_{A,\rm in}}{\sqrt{2\bar{\gamma}_{\rm in}}} - \alpha_A\right]\right\}\delta\gamma_{\rm in} \tag{117}$$
$$+ig_0 e^{-2im\phi_0}\left(\sqrt{2\bar{\gamma}_{\rm in}}\delta\tilde{s}a_{A,\rm in} + \sqrt{2\gamma_l}\delta\tilde{a}_{A,l}\right) + \left[\bar{\gamma} + i\left(\omega - g_{\rm sig} - \Delta\right)\right]\left(\sqrt{2\bar{\gamma}_{\rm in}}\tilde{a}_{C,\rm in} + \sqrt{2\gamma_l}\delta\tilde{a}_{C,l}\right)$$
$$= \left\{i\alpha_C\left[\bar{\gamma} + i\left(\omega - g_{\rm sig} - \Delta\right)\right] - \alpha_A g_0 e^{-2im\phi_0}\right\}g_{\rm therm}^{\rm self} \tag{118}$$
$$+i\alpha_A\left[\bar{\gamma} + i\left(\omega - g_{\rm sig} - \Delta\right)\right]R_{\rm therm}^{\rm cross} + \alpha_A\left[\bar{\gamma} + i\left(\omega - g_{\rm sig} - \Delta\right)\right]I_{\rm therm}^{\rm cross} \tag{119}$$
$$-\left\{\alpha_C\left[\bar{\gamma} + i\left(\omega - g_{\rm sig} - \Delta\right)\right] - ig_0 e^{-2im\phi_0}\left[\frac{\alpha_{A,\rm in}}{\sqrt{2\bar{\gamma}_{\rm in}}} - \alpha_A\right]\right\}\delta\gamma_{\rm in} \tag{120}$$
$$+ig_0 e^{-2im\phi_0}\left(\sqrt{2\bar{\gamma}_{\rm in}}\delta\tilde{a}_{A,\rm in} + \sqrt{2\gamma_l}\delta\tilde{a}_{A,l}\right) + \left[\bar{\gamma} + i\left(\omega - g_{\rm sig} - \Delta\right)\right]\left(\sqrt{2\bar{\gamma}_{\rm in}}\delta\tilde{a}_{C,\rm in} + \sqrt{2\gamma_l}\delta\tilde{a}_{C,l}\right)$$

where we have neglected product terms of g_0 and α_C since $g_0 \ll \bar{\gamma}$ and $\alpha_C \ll \alpha_A$.

The output field fluctuations $\delta\tilde{a}_{C,out}$ can be related to the input through $\delta\hat{a}_{C,\rm out} = \delta\tilde{a}_{C,\rm in} - \sqrt{2\bar{\gamma}_{\rm in}}\delta\tilde{a}_C - \alpha_C/\sqrt{2\bar{\gamma}_{\rm in}}\delta\gamma_{\rm in}$. Defining

$$\delta\tilde{a}_{C,\rm out} = Ag_{\rm therm}^{\rm self\,\prime} + BR_{\rm therm}^{\rm cross\,\prime} + CI_{\rm therm}^{\rm cross\,\prime} + D\delta\gamma_{\rm in}' + E\delta\tilde{a}_{A,\rm in} + F\delta\tilde{a}_{A,l} + G\delta\tilde{a}_{C,\rm in} + H\delta\tilde{a}_{C,l}, \tag{121}$$

we then have

$$
\begin{aligned}
A &= -\sqrt{2\bar{\gamma}_{\rm in}}\bar{\gamma}\frac{\left\{i\alpha_C\left[\bar{\gamma}+i\left(\omega-g_{\rm sig}-\Delta\right)\right]-\alpha_A g_0 e^{-2im\phi_0}\right\}}{\left[\bar{\gamma}+i\left(\omega-g_{\rm sig}-\Delta\right)\right]^2} && (122)\\
B &= -\frac{i\bar{\gamma}\alpha_A\sqrt{2\bar{\gamma}_{\rm in}}}{\bar{\gamma}+i\left(\omega-g_{\rm sig}-\Delta\right)} && (123)\\
C &= -\frac{\bar{\gamma}\alpha_A\sqrt{2\bar{\gamma}_{\rm in}}}{\bar{\gamma}+i\left(\omega-g_{\rm sig}-\Delta\right)} && (124)\\
D &= \bar{\gamma}\frac{\sqrt{2\bar{\gamma}_{\rm in}}\alpha_C\left[\bar{\gamma}+i\left(\omega-g_{\rm sig}-\Delta\right)\right]-ig_0e^{-2im\phi_0}\left(\alpha_{A,\rm in}-\sqrt{2\bar{\gamma}_{\rm in}}\alpha_A\right)}{\left[\bar{\gamma}+i\left(\omega-g_{\rm sig}-\Delta\right)\right]^2}-\frac{\bar{\gamma}\alpha_C}{\sqrt{2\bar{\gamma}_{\rm in}}} && (125)\\
E &= -\frac{2i\bar{\gamma}_{\rm in}g_0e^{-2im\phi_0}}{\left[\bar{\gamma}+i\left(\omega-g_{\rm sig}-\Delta\right)\right]^2}\approx-\frac{2i\eta g_0'e^{-2im\phi_0}}{[1+i(\omega'-\Delta')]^2} && (126)\\
F &= -\frac{2i\sqrt{\bar{\gamma}_{\rm in}\gamma_l}g_0e^{-2im\phi_0}}{\left[\bar{\gamma}+i\left(\omega-g_{\rm sig}-\Delta\right)\right]^2}\approx-\frac{2i\sqrt{\eta(1-\eta)}g_0'e^{-2im\phi_0}}{(1-i\Delta')^2} && (127)\\
G &= 1-\frac{2\bar{\gamma}_{\rm in}}{\bar{\gamma}+i\left(\omega-g_{\rm sig}-\Delta\right)}\approx 1-\frac{2\eta}{1-i\Delta'} && (128)\\
H &= -\frac{2\sqrt{\bar{\gamma}_{\rm in}\gamma_l}}{\bar{\gamma}+i\left(\omega-g_{\rm sig}-\Delta\right)}\approx-\frac{2\sqrt{\eta(1-\eta)}}{1-i\Delta'}, && (129)
\end{aligned}
$$

where in the expressions for E-H we have moved into dimensionless units, and made the approximation that $\{\omega, g_{\rm sig}\} \ll \bar{\gamma}$. In E we retain the ω' dependence since, latter in the calculation the other terms will cancel, leaving it as the dominant term. To simplify A-D we must substitute in for α_A and α_C.

$$
\begin{aligned}
A &= \frac{2\bar{\gamma}_{\rm in}\bar{\gamma}g_0e^{-2im\phi_0}}{(\bar{\gamma}-i\Delta)^2}\frac{\left\{\left[\bar{\gamma}+i\left(\omega-g_{\rm sig}-\Delta\right)\right]+\left[\bar{\gamma}-i\Delta-\frac{g_0^2}{(\bar{\gamma}-i\Delta)}\right]\right\}}{\left[\bar{\gamma}+i\left(\omega-g_{\rm sig}-\Delta\right)\right]^2}\alpha_{A,\rm in} && (130)\\
&\approx 2\bar{\gamma}_{\rm in}\bar{\gamma}g_0e^{-2im\phi_0}\left(\frac{2\bar{\gamma}+i(\omega-2\Delta)}{(\bar{\gamma}-i\Delta)^2\left[\bar{\gamma}+i(\omega-\Delta)\right]^2}\right)\alpha_{A,\rm in} && (131)\\
&= 2\eta g_0'e^{-2im\phi_0}\left(\frac{2+i(\omega'-2\Delta')}{(1-i\Delta')^2\left[1+i(\omega'-\Delta')\right]^2}\right)\alpha_{A,\rm in} && (132)\\
&\approx \frac{4\eta g_0'e^{-2im\phi_0}}{(1-i\Delta')^3}\alpha_{A,\rm in}, && (133)
\end{aligned}
$$

taking $\{g_{\rm sig}, \omega\} \ll \bar{\gamma}$, and $g_0^2 \ll \bar{\gamma}^2$.

$$
\begin{aligned}
B &= -\frac{2i\bar{\gamma}\bar{\gamma}_{\rm in}}{(\bar{\gamma}-i\Delta)^2}\left[1-\frac{g_0^2}{(\bar{\gamma}-i\Delta)^2}\right]\alpha_{A,\rm in} && (134)\\
&= -2i\eta\left(\frac{(1-i\Delta')^2-{g_0'}^2}{(1-i\Delta')^4}\right)\alpha_{A,\rm in} && (135)
\end{aligned}
$$

$$C = -iB \qquad (136)$$

$$D = \frac{i\bar{\gamma}g_0 e^{-2im\phi_0}}{(\bar{\gamma}-i\Delta)^2}\left(\frac{2\bar{\gamma}_{\rm in}\alpha_{A,\rm in}}{\bar{\gamma}-i\Delta} - 2\alpha_{A,\rm in} + \sqrt{2\bar{\gamma}_{\rm in}}\alpha_A\right) \tag{137}$$

$$= \frac{i\bar{\gamma}g_0 e^{-2im\phi_0}}{(\bar{\gamma}-i\Delta)^3}\left(2\bar{\gamma}_{\rm in}\alpha_{A,\rm in} - 2\alpha_{A,\rm in}(\bar{\gamma}-i\Delta) + 2\bar{\gamma}_{\rm in}\left[1 - \frac{g_0^2}{(\bar{\gamma}-i\Delta)^2}\right]\alpha_{A,\rm in}\right) \tag{138}$$

$$= \frac{2i\bar{\gamma}g_0 e^{-2im\phi_0}}{(\bar{\gamma}-i\Delta)^3}\left[2\bar{\gamma}_{\rm in} - \bar{\gamma} + i\Delta\right]\alpha_{A,\rm in} \tag{139}$$

$$= \frac{2ig_0' e^{-2im\phi_0}}{(1-i\Delta')^3}\left[2\eta - 1 + i\Delta'\right]\alpha_{A,\rm in} \tag{140}$$

The output field can now be determined as

$$\tilde{a}_{C,\rm out} = \alpha_{C,\rm out} + \delta\tilde{a}_{C,\rm out} \tag{141}$$

$$= -\frac{2i\eta}{(1-i\Delta')^2}\left(g'_{\rm sig}e^{-2im\phi_{\rm sig}} + g_0' e^{-2im\phi_0}\right)\alpha_{A,\rm in} + \delta\tilde{a}_{C,\rm out} \tag{142}$$

Let us determine the signal that is received on direct detection of this field, i.e.

$$i = \tilde{a}^\dagger_{C,\rm out}(-\omega)\tilde{a}_{C,\rm out}(\omega) \tag{143}$$

$$= \frac{4\eta^2}{\left(1+\Delta'^2\right)^2}\left|g'_{\rm sig}e^{2im\phi_{\rm sig}} + g_0' e^{2im\phi_0}\right|^2\alpha^2_{A,\rm in} - \frac{2i\eta}{(1-i\Delta')^2}g_0' e^{-2im\phi_0}\alpha_{A,\rm in}\delta\tilde{a}^\dagger_{C,\rm out} + \frac{2i\eta}{(1+i\Delta')^2}g_0' e^{2im\phi_0}\alpha_{A,\rm in}\delta\tilde{a}_{C,\rm out} \tag{144}$$

$$= \frac{4g_0'\eta^2\alpha^2_{A,\rm in}}{\left(1+\Delta'^2\right)^2}\left[g_0' + g'_{\rm sig}\left(e^{-2im(\phi_{\rm sig}-\phi_0)} + e^{2im(\phi_{\rm sig}-\phi_0)}\right)\right] + 2i\eta g_0'\alpha_{A,\rm in}\left(\frac{e^{2im\phi_0}}{(1+i\Delta')^2}\delta\tilde{a}_{C,\rm out} - \frac{e^{-2im\phi_0}}{(1-i\Delta')^2}\delta\tilde{a}^\dagger_{C,\rm out}\right) \tag{145}$$

$$= \frac{4g_0'\eta^2\alpha^2_{A,\rm in}}{\left(1+\Delta'^2\right)^2}\left[g_0' + 2g'_{\rm sig}\cos 2m(\phi_{\rm sig}-\phi_0)\right] + 2i\eta g_0'\alpha_{A,\rm in}\left(\frac{e^{2im\phi_0}}{(1+i\Delta')^2}\delta\tilde{a}_{C,\rm out} - \frac{e^{-2im\phi_0}}{(1-i\Delta')^2}\delta\tilde{a}^\dagger_{C,\rm out}\right) \tag{146}$$

neglecting the noise product term $\delta\tilde{a}^\dagger_{C,\rm out}(-\omega)\delta\tilde{a}_{C,\rm out}(\omega)$, and, since the signal is small the product terms of signal $g'_{\rm sig}$ and noise $\delta\tilde{a}_{C,\rm out}$, and the signal product term ${g'_{\rm sig}}^2$.

The mean measured signal is then

$$\langle i\rangle = \frac{4g_0'\eta^2\alpha^2_{A,\rm in}}{\left(1+\Delta'^2\right)^2}\left[g_0' + 2g'_{\rm sig}\cos 2m(\phi_{\rm sig}-\phi_0)\right] \tag{147}$$

Rearranging this expression in terms of $g'_{\rm sig}$ we have

$$g'_{\rm sig} = \frac{1}{2\cos 2m(\phi_{\rm sig}-\phi_0)}\left[\frac{\left(1+\Delta'^2\right)^2}{4g_0'\eta^2\alpha^2_{A,\rm in}}\langle i\rangle - g_0'\right], \tag{148}$$

such that, based on the measurement i an estimate of $g'_{\rm sig}$ may be formed as

$$g^{\rm est\,\prime}_{\rm sig} = g'_{\rm sig} = \frac{1}{2\cos 2m(\phi_{\rm sig}-\phi_0)}\left[\frac{\left(1+\Delta'^2\right)^2}{4g_0'\eta^2\alpha^2_{A,\rm in}}i - g_0'\right]. \tag{149}$$

The uncertainty in this estimate is

$$\left\langle\left|\delta g^{\rm est\,\prime}_{\rm sig}\right|^2\right\rangle_{\rm back-scatter} = \frac{\left(1+\Delta'^2\right)^4}{64{g_0'}^2\eta^4\alpha^4_{A,\rm in}\cos^2 2m(\phi_{\rm sig}-\phi_0)}\left\langle|\delta i|^2\right\rangle \tag{150}$$

$$\approx \frac{\left\langle|\delta i|^2\right\rangle}{64{g_0'}^2\eta^4\alpha^4_{A,\rm in}\cos^2 2m(\phi_{\rm sig}-\phi_0)} \tag{151}$$

Now we must find $\left\langle |\delta i|^2 \right\rangle$

$$\left\langle |\delta i|^2 \right\rangle = 4\eta^2 {g_0'}^2 \alpha_{A,\text{in}}^2 \left\langle \left| \frac{e^{2im\phi_0}}{(1+i\Delta')^2} \delta\tilde{a}_{C,\text{out}}(\omega) - \frac{e^{-2im\phi_0}}{(1-i\Delta')^2} \delta\tilde{a}^\dagger_{C,\text{out}}(-\omega) \right|^2 \right\rangle \tag{152}$$

$$= \frac{4\eta^2 {g_0'}^2 \alpha_{A,\text{in}}^2}{\left(1+\Delta'^2\right)^2} \left\langle \left| e^{2im\breve{\phi}} \delta\tilde{a}_{C,\text{out}}(\omega) - e^{-2im\breve{\phi}} \delta\tilde{a}^\dagger_{C,\text{out}}(-\omega) \right|^2 \right\rangle, \tag{153}$$

where for conciseness we have combined the phase shift due to detuning with that due to g_0', making the definitions $(1+\Delta'^2)/(1+i\Delta')^2 = e^{2im\phi_\Delta}$, and $\breve{\phi} = \phi_0 + \phi_\Delta$. In general the expectation value may be expanded in terms of a series of noise operators $\delta\tilde{a}_j$ each with coefficients A_j (in our case these coefficients are A through H, and the noise operators are those associated to each of A through H).

$$\left\langle \left| e^{2im\breve{\phi}} \delta\tilde{a}_{C,\text{out}}(\omega) - e^{-2im\breve{\phi}} \delta\tilde{a}^\dagger_{C,\text{out}}(-\omega) \right|^2 \right\rangle = \left\langle \left| e^{2im\breve{\phi}} \sum_j A_j(\omega)\delta\tilde{a}_j(\omega) - e^{-2im\breve{\phi}} \sum_j A_j^*(-\omega)\delta\tilde{a}_j^\dagger(-\omega) \right|^2 \right\rangle \tag{154}$$

$$= \sum_j \left\langle \left| e^{2im\breve{\phi}} A_j(\omega)\delta\tilde{a}_j(\omega) - e^{-2im\breve{\phi}} A_j^*(-\omega)\delta\tilde{a}_j^\dagger(-\omega) \right|^2 \right\rangle \tag{155}$$

where we have assumed, as is the relevant case here, that all noise sources are uncorrelated. It therefore only remains to calculate the contributions to the variance from each noise source.

$$\left\langle \left| e^{2im\breve{\phi}} A {g^{\text{self}}_{\text{therm}}}' - e^{-2im\breve{\phi}} A^* {g^{\text{self}}_{\text{therm}}}'^* \right|^2 \right\rangle = \left| e^{2im\breve{\phi}} A - e^{-2im\breve{\phi}} A^* \right|^2 \left\langle {g^{\text{self}}_{\text{therm}}}'^2 \right\rangle \tag{156}$$

$$= \left| e^{2im\breve{\phi}} \frac{4\eta g_0' e^{-2im\phi_0}}{(1-i\Delta')^3} \alpha_{A,\text{in}} - e^{-2im\breve{\phi}} \frac{4\eta g_0' e^{2im\phi_0}}{(1+i\Delta')^3} \alpha_{A,\text{in}} \right|^2 \left\langle {g^{\text{self}}_{\text{therm}}}'^2 \right\rangle \tag{157}$$

$$= 16\eta^2 {g_0'}^2 \alpha_{A,\text{in}}^2 \left| \frac{1+\Delta'^2}{(1+\Delta'^2)^2(1-i\Delta')} - \frac{1+\Delta'^2}{(1+\Delta'^2)^2(1+i\Delta')} \right|^2 \left\langle {g^{\text{self}}_{\text{therm}}}'^2 \right\rangle \tag{158}$$

$$= \frac{16\eta^2 {g_0'}^2 \alpha_{A,\text{in}}^2}{(1+\Delta'^2)^2} \left| \frac{1}{1-i\Delta'} - \frac{1}{1+i\Delta'} \right|^2 \left\langle {g^{\text{self}}_{\text{therm}}}'^2 \right\rangle \tag{159}$$

$$= 64\eta^2 {g_0'}^2 \alpha_{A,\text{in}}^2 \frac{\Delta'^2}{(1+\Delta'^2)^4} \left\langle {g^{\text{self}}_{\text{therm}}}'^2 \right\rangle \tag{160}$$

where we've used the fact that $e^{2im\breve{\phi}} e^{-2im\phi_0} = e^{2im\phi_\Delta} = (1+\Delta'^2)/(1+i\Delta')^2$. From this, we see that, in contrast to sensing using a transmitted phase shift, when using backscattered light, the self-thermorefractive noise is greatly suppressed. If the cavity is put exactly on resonance $\Delta = 0$, then self-thermorefractive noise is exactly eliminated.

$$\left\langle\left|e^{2im\breve{\phi}}BR^{\text{cross}\,\prime}_{\text{therm}} - e^{-2im\breve{\phi}}B^*R^{\text{cross}\,\prime *}_{\text{therm}}\right|^2\right\rangle = \left|e^{2im\breve{\phi}}B - e^{-2im\breve{\phi}}B^*\right|^2 \left\langle R^{\text{cross}\,\prime 2}_{\text{therm}}\right\rangle \tag{161}$$

$$= \left|-e^{2im\breve{\phi}}2i\eta\left(\frac{(1-i\Delta')^2 - g_0'^2}{(1-i\Delta')^4}\right)\alpha_{A,\text{in}} - e^{-2im\breve{\phi}}2i\eta\left(\frac{(1+i\Delta')^2 - g_0'^2}{(1+i\Delta')^4}\right)\alpha_{A,\text{in}}\right|^2 \left\langle R^{\text{cross}\,\prime 2}_{\text{therm}}\right\rangle$$

$$= 4\eta^2\alpha^2_{A,\text{in}}\left|e^{2im\breve{\phi}}\left(\frac{(1-i\Delta')^2 - g_0'^2}{(1-i\Delta')^4}\right) + e^{-2im\breve{\phi}}\left(\frac{(1+i\Delta')^2 - g_0'^2}{(1+i\Delta')^4}\right)\right|^2 \left\langle R^{\text{cross}\,\prime 2}_{\text{therm}}\right\rangle$$

$$= 4\eta^2\alpha^2_{A,\text{in}}\left|e^{2im\breve{\phi}}\left(\frac{(1+\Delta'^2)e^{2im\phi_\Delta}}{\left(1+\Delta'^2\right)^2 e^{4im\phi_\Delta}}\right) + e^{-2im\breve{\phi}}\left(\frac{(1+\Delta'^2)e^{-2im\phi_\Delta}}{\left(1+\Delta'^2\right)^2 e^{-4im\phi_\Delta}}\right)\right|^2 \left\langle R^{\text{cross}\,\prime 2}_{\text{therm}}\right\rangle$$

$$= \frac{4\eta^2\alpha^2_{A,\text{in}}}{\left(1+\Delta'^2\right)^2}\left|e^{2im\phi_0} + e^{-2im\phi_0}\right|^2 \left\langle R^{\text{cross}\,\prime 2}_{\text{therm}}\right\rangle$$

$$= \frac{16\eta^2\alpha^2_{A,\text{in}}}{\left(1+\Delta'^2\right)^2}\cos^2 2m\phi_0 \left\langle R^{\text{cross}\,\prime 2}_{\text{therm}}\right\rangle \tag{162}$$

$$\approx 16\eta^2\alpha^2_{A,\text{in}}\cos^2 2m\phi_0 \left\langle R^{\text{cross}\,\prime 2}_{\text{therm}}\right\rangle, \tag{163}$$

where we have taken $g_0' \ll 1$. From this we see that the cross-thermorefractive noise does contribute to the measured noise in the back-scatter detection protocol, so it is not possible to entirely avoid thermo-refractive noise.

$$\left\langle\left|e^{2im\breve{\phi}}CI^{\text{cross}\,\prime}_{\text{therm}} - e^{-2im\breve{\phi}}C^*I^{\text{cross}\,\prime *}_{\text{therm}}\right|^2\right\rangle = \left|e^{2im\breve{\phi}}C - e^{-2im\breve{\phi}}C^*\right|^2 \left\langle I^{\text{cross}\,\prime 2}_{\text{therm}}\right\rangle \tag{164}$$

$$= \left|ie^{2im\breve{\phi}}B + ie^{-2im\breve{\phi}}B^*\right|^2 \left\langle I^{\text{cross}\,\prime 2}_{\text{therm}}\right\rangle \tag{165}$$

$$= \left|e^{2im\breve{\phi}}B + e^{-2im\breve{\phi}}B^*\right|^2 \left\langle R^{\text{cross}\,\prime 2}_{\text{therm}}\right\rangle \tag{166}$$

$$= \frac{4\eta^2\alpha^2_{A,\text{in}}}{\left(1+\Delta'^2\right)^2}\left|e^{2im\phi_0} - e^{-2im\phi_0}\right|^2 \left\langle R^{\text{cross}\,\prime 2}_{\text{therm}}\right\rangle$$

$$= \frac{16\eta^2\alpha^2_{A,\text{in}}}{\left(1+\Delta'^2\right)^2}\sin^2 2m\phi_0 \left\langle R^{\text{cross}\,\prime 2}_{\text{therm}}\right\rangle \tag{167}$$

$$\approx 16\eta^2\alpha^2_{A,\text{in}}\sin^2 2m\phi_0 \left\langle R^{\text{cross}\,\prime 2}_{\text{therm}}\right\rangle, \tag{168}$$

where we've used the fact that $\left\langle I^{\text{cross}\,\prime 2}_{\text{therm}}\right\rangle = \left\langle R^{\text{cross}\,\prime 2}_{\text{therm}}\right\rangle$ (Eq. (43)).

From Eqs. (169) and (167) it can be seen that the total contribution of thermorefractive noise to the measured photocurrent is

$$\left\langle\left|e^{2im\breve{\phi}}BR^{\text{cross}\,\prime}_{\text{therm}} - e^{-2im\breve{\phi}}B^*R^{\text{cross}\,\prime *}_{\text{therm}}\right|^2\right\rangle + \left\langle\left|e^{2im\breve{\phi}}CI^{\text{cross}\,\prime}_{\text{therm}} - e^{-2im\breve{\phi}}C^*I^{\text{cross}\,\prime *}_{\text{therm}}\right|^2\right\rangle = \frac{16\eta^2\alpha^2_{A,\text{in}}}{\left(1+\Delta'^2\right)^2}\left\langle R^{\text{cross}\,\prime 2}_{\text{therm}}\right\rangle \tag{169}$$

$$\approx 16\eta^2\alpha^2_{A,\text{in}}\left\langle R^{\text{cross}\,\prime 2}_{\text{therm}}\right\rangle. \tag{170}$$

$$\left\langle \left| e^{2im\breve{\phi}} D\delta\gamma'_{\rm in} - e^{-2im\breve{\phi}} D^* \delta\gamma'^{\,*}_{\rm in} \right|^2 \right\rangle = \left| e^{2im\breve{\phi}} D - e^{-2im\breve{\phi}} D^* \right|^2 \left\langle \delta\gamma'^{\,2}_{\rm in} \right\rangle \tag{171}$$

$$= \left| e^{2im\breve{\phi}} \frac{2ig'_0 e^{-2im\phi_0}}{(1-i\Delta')^3} \left[2\eta - 1 + i\Delta'\right] \alpha_{A,\rm in} + e^{-2im\breve{\phi}} \frac{2ig'_0 e^{2im\phi_0}}{(1+i\Delta')^3} \left[2\eta - 1 - i\Delta'\right] \alpha_{A,\rm in} \right|^2 \left\langle \delta\gamma'^{\,2}_{\rm in} \right\rangle$$

$$= 4g'^{\,2}_0 \alpha^2_{A,\rm in} \left| \frac{e^{2im\phi_\Delta}}{(1-i\Delta')^3} \left[2\eta - 1 + i\Delta'\right] + \frac{e^{-2im\phi_\Delta}}{(1+i\Delta')^3} \left[2\eta - 1 - i\Delta'\right] \right|^2 \left\langle \delta\gamma'^{\,2}_{\rm in} \right\rangle$$

$$= 4g'^{\,2}_0 \alpha^2_{A,\rm in} \left| \frac{1+\Delta'^2}{(1-i\Delta')^3 (1+i\Delta')^2} \left[2\eta - 1 + i\Delta'\right] + \frac{1+\Delta'^2}{(1+i\Delta')^3 (1-i\Delta')^2} \left[2\eta - 1 - i\Delta'\right] \right|^2 \left\langle \delta\gamma'^{\,2}_{\rm in} \right\rangle$$

$$= \frac{4g'^{\,2}_0 \alpha^2_{A,\rm in}}{(1+\Delta'^2)^2} \left| \frac{2\eta - 1 + i\Delta'}{(1-i\Delta')} + \frac{2\eta - 1 - i\Delta'}{(1+i\Delta')} \right|^2 \left\langle \delta\gamma'^{\,2}_{\rm in} \right\rangle$$

$$= \frac{4g'^{\,2}_0 \alpha^2_{A,\rm in}}{(1+\Delta'^2)^2} \left| \frac{2\eta - 1 + 2i\eta\Delta' - \Delta'^2}{1+\Delta'^2} + \frac{2\eta - 1 - 2i\eta\Delta' - \Delta'^2}{1+\Delta'^2} \right|^2 \left\langle \delta\gamma'^{\,2}_{\rm in} \right\rangle$$

$$= 16 g'^{\,2}_0 \alpha^2_{A,\rm in} \left[\frac{2\eta - 1 - \Delta'^2}{(1+\Delta'^2)^2} \right]^2 \left\langle \delta\gamma'^{\,2}_{\rm in} \right\rangle$$

$$\approx 16 g'^{\,2}_0 \alpha^2_{A,\rm in} (2\eta - 1)^2 \left\langle \delta\gamma'^{\,2}_{\rm in} \right\rangle$$

This expression is identical to the noise term due to input coupling fluctuations in the direct measurement case (see Eq. 91), with the exception that here $\Delta' = \Delta'_0 + g'_0$ is replaced with simply g'_0. Hence, a disadvantage of the backscatter scheme is that input coupling noise cannot be removed by simply ensuring the light in on resonance with the cavity. However, it should be noticed, that this noise source may be arbitrarily suppressed in principle by sitting close to critical coupling where $\eta = 0.5$.

$$\left\langle \left| e^{2im\breve{\phi}} E(\omega) \delta\tilde{a}_{A,\rm in} - e^{-2im\breve{\phi}} E^*(-\omega) \delta\tilde{a}_{A,\rm in}^{\dagger} \right|^2 \right\rangle = \frac{1}{4} \left\langle \left| e^{2im\breve{\phi}} E \left(\delta\tilde{X}_{A,\rm in} + i\delta\tilde{Y}_{A,\rm in} \right) - e^{-2im\breve{\phi}} E^* \left(\delta\tilde{X}_{A,\rm in} - i\delta\tilde{Y}_{A,\rm in} \right) \right|^2 \right\rangle \tag{172}$$

$$= \frac{1}{4} \left\langle \left| e^{2im\breve{\phi}} E \delta\tilde{X}_{A,\rm in} - e^{-2im\breve{\phi}} E^* \delta\tilde{X}_{A,\rm in} \right|^2 \right\rangle + \frac{1}{4} \left\langle \left| e^{2im\breve{\phi}} E \delta\tilde{Y}_{A,\rm in} + e^{-2im\breve{\phi}} E^* \delta\tilde{Y}_{A,\rm in} \right|^2 \right\rangle$$

$$= \frac{1}{4} \left| e^{2im\breve{\phi}} E(\omega) - e^{-2im\breve{\phi}} E^*(-\omega) \right|^2 \left\langle \delta\tilde{X}^2_{A,\rm in} \right\rangle + \frac{1}{4} \left| e^{2im\breve{\phi}} E(\omega) + e^{-2im\breve{\phi}} E^*(-\omega) \right|^2 \left\langle \delta\tilde{Y}^2_{A,\rm in} \right\rangle .$$

$$\left| e^{2im\breve{\phi}} E(\omega) - e^{-2im\breve{\phi}} E^*(-\omega) \right|^2 = \left| e^{2im\breve{\phi}} \frac{2i\eta g'_0 e^{-2im\phi_0}}{\left[1 + i(\omega' - \Delta')\right]^2} + e^{-2im\breve{\phi}} \frac{2i\eta g'_0 e^{2im\phi_0}}{\left[1 + i(\omega' + \Delta')\right]^2} \right|^2 \tag{173}$$

$$= 4\eta^2 g'^{\,2}_0 \left(1+\Delta'^2\right)^2 \left| \frac{1}{(1+i\Delta')^2 \left[1 + i(\omega' - \Delta')\right]^2} + \frac{1}{(1-i\Delta')^2 \left[1 + i(\omega' + \Delta')\right]^2} \right|^2 \tag{174}$$

$$= 4\eta^2 g'^{\,2}_0 \left(1+\Delta'^2\right)^2 \left| \frac{(1-i\Delta')^2 \left[1 + i(\omega' + \Delta')\right]^2 + (1+i\Delta')^2 \left[1 + i(\omega' - \Delta')\right]^2}{(1+\Delta'^2)^2 \left[1 + i(\omega' - \Delta')\right]^2 \left[1 + i(\omega' + \Delta')\right]^2} \right|^2 \tag{175}$$

$$= 4\eta^2 g'^{\,2}_0 \frac{\left| \left(1 - 2i\Delta' - \Delta'^2\right) \left[1 + 2i(\omega' + \Delta') - (\omega' + \Delta')^2\right] + \left(1 + 2i\Delta' - \Delta'^2\right) \left[1 + 2i(\omega' - \Delta') - (\omega' - \Delta')^2\right] \right|^2}{(1+\Delta'^2)^2 \left[1 + (\omega' - \Delta')^2\right]^2 \left[1 + (\omega' + \Delta')^2\right]^2}$$

$$= 16\eta^2 g'^{\,2}_0 \frac{\left(1 - \omega'^2 + 2\Delta'^2 + \Delta'^2\omega'^2 + \Delta'^4\right)^2 + 4\omega'^2 \left(1+\Delta'^2\right)^2}{(1+\Delta'^2)^2 \left[1 + (\omega' - \Delta')^2\right]^2 \left[1 + (\omega' + \Delta')^2\right]^2} \tag{176}$$

$$\approx 16\eta^2 g'^{\,2}_0 \frac{\left(1 + 2\Delta'^2 + \Delta'^4\right)^2}{(1+\Delta'^2)^2 (1+\Delta'^2)^2 (1+\Delta'^2)^2} \tag{177}$$

$$= \frac{16\eta^2 g'^{\,2}_0}{(1+\Delta'^2)^2} \tag{178}$$

$$\approx 16\eta^2 g'^{\,2}_0, \tag{179}$$

where at Eq. (177) we have made the approximation $\omega' \ll 1$, and at Eq. (179) we have made the approximation $\Delta' \ll 1$. Similarly, for the second term in E we find

$$\begin{aligned}
\left|e^{2im\breve{\phi}}E(\omega)+e^{-2im\breve{\phi}}E^*(-\omega)\right|^2 &= \left|e^{2im\breve{\phi}}\frac{2i\eta g_0' e^{-2im\phi_0}}{[1+i(\omega'-\Delta')]^2}-e^{-2im\breve{\phi}}\frac{2i\eta g_0' e^{2im\phi_0}}{[1+i(\omega'+\Delta')]^2}\right|^2 && (180)\\
&= 4\eta^2 {g_0'}^2\left(1+\Delta'^2\right)^2\left|\frac{1}{(1+i\Delta')^2[1+i(\omega'-\Delta')]^2}-\frac{1}{(1-i\Delta')^2[1+i(\omega'+\Delta')]^2}\right|^2 && (181)\\
&= 4\eta^2 {g_0'}^2\left(1+\Delta'^2\right)^2\left|\frac{(1-i\Delta')^2[1+i(\omega'+\Delta')]^2-(1+i\Delta')^2[1+i(\omega'-\Delta')]^2}{(1+\Delta'^2)^2[1+i(\omega'-\Delta')]^2[1+i(\omega'+\Delta')]^2}\right|^2 && (182)\\
&= 4\eta^2 {g_0'}^2\frac{\left|\left(1-2i\Delta'-\Delta'^2\right)\left[1+2i(\omega'+\Delta')-(\omega'+\Delta')^2\right]-\left(1+2i\Delta'-\Delta'^2\right)\left[1+2i(\omega'-\Delta')-(\omega'-\Delta')^2\right]\right|^2}{(1+\Delta'^2)^2\left[1+(\omega'-\Delta')^2\right]^2\left[1+(\omega'+\Delta')^2\right]^2}\\
&= 64\eta^2 {g_0'}^2\frac{\omega'^2\Delta'^2\left(1+\omega'^2\right)^2+\Delta'^2\omega'^4}{(1+\Delta'^2)^2\left[1+(\omega'-\Delta')^2\right]^2\left[1+(\omega'+\Delta')^2\right]^2} && (183)\\
&= 64\eta^2 {g_0'}^2\omega'^2\Delta'^2\frac{\left(1+\omega'^2\right)^2+\omega'^2}{(1+\Delta'^2)^2\left[1+(\omega'-\Delta')^2\right]^2\left[1+(\omega'+\Delta')^2\right]^2} && (184)\\
&\approx \frac{64\eta^2 {g_0'}^2\omega'^2\Delta'^2}{(1+\Delta'^2)^6} && (185)\\
&\approx 64\eta^2 {g_0'}^2\omega'^2\Delta'^2. && (186)
\end{aligned}$$

We therefore find

$$\begin{aligned}
\left\langle\left|e^{2im\breve{\phi}}E\delta\tilde{a}_{A,\mathrm{in}}-e^{-2im\breve{\phi}}E^*\delta\tilde{a}_{A,\mathrm{in}}^{\dagger}\right|^2\right\rangle &= \frac{4\eta^2{g_0'}^2}{(1+\Delta'^2)^2}\left\langle\delta\tilde{X}^2_{A,\mathrm{in}}\right\rangle+\frac{16\eta^2{g_0'}^2\omega'^2\Delta'^2}{(1+\Delta'^2)^6}\left\langle\delta\tilde{Y}^2_{A,\mathrm{in}}\right\rangle && (187)\\
&\approx 4\eta^2{g_0'}^2\left\langle\delta\tilde{X}^2_{A,\mathrm{in}}\right\rangle+16\eta^2{g_0'}^2\omega'^2\Delta'^2\left\langle\delta\tilde{Y}^2_{A,\mathrm{in}}\right\rangle && (188)
\end{aligned}$$

Substituting for the amplitude and phase noise of the incident field from Eqs. (52) and (53) we have

$$\begin{aligned}
\left\langle\left|e^{2im\breve{\phi}}E\delta\tilde{a}_{A,\mathrm{in}}-e^{-2im\breve{\phi}}E^*\delta\tilde{a}_{A,\mathrm{in}}^{\dagger}\right|^2\right\rangle &= \frac{4\eta^2{g_0'}^2}{(1+\Delta'^2)^2}\left(1+V_{\mathrm{RIN}}(\omega)\alpha^2_{A,\mathrm{in}}\right)+\frac{16\eta^2{g_0'}^2\omega'^2\Delta'^2}{(1+\Delta'^2)^6}\left(1+V_{\zeta}(\omega)\alpha^2_{A,\mathrm{in}}\right) && (189)\\
&\approx \frac{4\eta^2{g_0'}^2}{(1+\Delta'^2)^2}\left[1+V_{\mathrm{RIN}}(\omega)\alpha^2_{A,\mathrm{in}}+\frac{4\omega'^2\Delta'^2}{(1+\Delta'^2)^4}V_{\zeta}(\omega)\alpha^2_{A,\mathrm{in}}\right] && (190)\\
&\approx 4\eta^2{g_0'}^2\left[1+\alpha^2_{A,in}\left(V_{\mathrm{RIN}}(\omega)+4\omega'^2\Delta'^2V_{\zeta}(\omega)\right)\right] && (191)
\end{aligned}$$

We see that there is a fundamental noise floor due to shot noise (the 1), whilst laser phase noise is suppressed relative to intensity noise by a factor given by $4\omega'^2\Delta'^2$.

$$\left\langle \left| e^{2im\check{\phi}} F \delta\tilde{a}_{A,l} - e^{-2im\check{\phi}} F^* \delta\tilde{a}_{A,l}{}^\dagger \right|^2 \right\rangle = \frac{1}{4} \left\langle \left| e^{2im\check{\phi}} F \left(\delta\tilde{X}_{A,l} + i\delta\tilde{Y}_{A,l} \right) - e^{-2im\check{\phi}} F^* \left(\delta\tilde{X}_{A,l} - i\delta\tilde{Y}_{A,l} \right) \right|^2 \right\rangle \tag{192}$$

$$= \frac{1}{4} \left\langle \left| e^{2im\check{\phi}} F \delta\tilde{X}_{A,l} - e^{-2im\check{\phi}} F^* \delta\tilde{X}_{A,l} \right|^2 \right\rangle + \frac{1}{4} \left\langle \left| e^{2im\check{\phi}} F \delta\tilde{Y}_{A,l} + e^{-2im\check{\phi}} F^* \delta\tilde{Y}_{A,l} \right|^2 \right\rangle \tag{193}$$

$$= \frac{1}{4} \left| e^{2im\check{\phi}} F - e^{-2im\check{\phi}} F^* \right|^2 \left\langle \delta\tilde{X}^2_{A,l} \right\rangle + \frac{1}{4} \left| e^{2im\check{\phi}} F + e^{-2im\check{\phi}} F^* \right|^2 \left\langle \delta\tilde{Y}^2_{A,l} \right\rangle \tag{194}$$

$$= \frac{4\eta(1-\eta){g'_0}^2}{(1+\Delta'^2)^2} \left\langle \delta\tilde{X}^2_{A,l} \right\rangle + \frac{16\eta(1-\eta){g'_0}^2\omega'^2\Delta'^2}{(1+\Delta'^2)^6} \left\langle \delta\tilde{Y}^2_{A,l} \right\rangle \tag{195}$$

$$= \frac{4\eta(1-\eta){g'_0}^2}{(1+\Delta'^2)^2} + \frac{16\eta(1-\eta){g'_0}^2\omega'^2\Delta'^2}{(1+\Delta'^2)^6} \tag{196}$$

$$= \frac{4\eta(1-\eta){g'_0}^2}{(1+\Delta'^2)^2} \left[1 + \frac{4\omega'^2\Delta'^2}{(1+\Delta'^2)^4} \right] \tag{197}$$

$$\approx 4\eta(1-\eta){g'_0}^2, \tag{198}$$

where to get to Eq. (195) we have used Eq. (187) combined with the fact that F is identical to E except for the substitution $\eta^2 \to \eta(1-\eta)$, and to get to Eq. (196) we have used the fact that the incident field entering the system through the cavity loss channels is in a vacuum state, and therefore $\left\langle \delta\tilde{X}^2_{A,l} \right\rangle = \left\langle \delta\tilde{Y}^2_{A,l} \right\rangle = 1$.

$$\left\langle \left| e^{2im\check{\phi}} G \delta\tilde{a}_{C,\mathrm{in}} - e^{-2im\check{\phi}} G^* \delta\tilde{a}_{C,\mathrm{in}}{}^\dagger \right|^2 \right\rangle = \frac{1}{4} \left\langle \left| e^{2im\check{\phi}} G \left(\delta\tilde{X}_{C,\mathrm{in}} + i\delta\tilde{Y}_{C,\mathrm{in}} \right) - e^{-2im\check{\phi}} G^* \left(\delta\tilde{X}_{C,\mathrm{in}} - i\delta\tilde{Y}_{C,\mathrm{in}} \right) \right|^2 \right\rangle \tag{199}$$

$$= \frac{1}{4} \left\langle \left| e^{2im\check{\phi}} G \delta\tilde{X}_{C,\mathrm{in}} - e^{-2im\check{\phi}} G^* \delta\tilde{X}_{C,\mathrm{in}} \right|^2 \right\rangle + \frac{1}{4} \left\langle \left| e^{2im\check{\phi}} G \delta\tilde{Y}_{C,\mathrm{in}} + e^{-2im\check{\phi}} G^* \delta\tilde{Y}_{C,\mathrm{in}} \right|^2 \right\rangle \tag{200}$$

$$= \frac{1}{4} \left| e^{2im\check{\phi}} G - e^{-2im\check{\phi}} G^* \right|^2 \left\langle \delta\tilde{X}^2_{C,\mathrm{in}} \right\rangle + \frac{1}{4} \left| e^{2im\check{\phi}} G + e^{-2im\check{\phi}} G^* \right|^2 \left\langle \delta\tilde{Y}^2_{C,\mathrm{in}} \right\rangle \tag{201}$$

$$= \frac{1}{4} \left| e^{2im\check{\phi}} G - e^{-2im\check{\phi}} G^* \right|^2 + \frac{1}{4} \left| e^{2im\check{\phi}} G + e^{-2im\check{\phi}} G^* \right|^2 \tag{202}$$

$$= \frac{1}{4} \left(2|G|^2 - 2|G|^2 + 2|G|^2 + 2|G|^2 \right) \tag{203}$$

$$= |G|^2 \tag{204}$$

$$= \left| 1 - \frac{2\eta}{1 - i\Delta'} \right|^2 \tag{205}$$

$$= \frac{(1-2\eta)^2 + \Delta'^2}{1+\Delta'^2} \tag{206}$$

$$\approx (1-2\eta)^2 + \Delta'^2 \tag{207}$$

where to arrive at Eq. (202) we have used the fact that the incident field entering the system through the input channel into mode C is in a vacuum state, and therefore $\left\langle \delta\tilde{X}^2_{C,\mathrm{in}} \right\rangle = \left\langle \delta\tilde{Y}^2_{C,\mathrm{in}} \right\rangle = 1$.

$$\left\langle \left| e^{2im\breve{\phi}} H \delta\tilde{a}_{C,l} - e^{-2im\breve{\phi}} H^* \delta\tilde{a}_{C,l}{}^{\dagger} \right|^2 \right\rangle = \frac{1}{4} \left\langle \left| e^{2im\breve{\phi}} H \left(\delta\tilde{X}_{C,l} + i\delta\tilde{Y}_{C,l} \right) - e^{-2im\breve{\phi}} H^* \left(\delta\tilde{X}_{C,l} - i\delta\tilde{Y}_{C,l} \right) \right|^2 \right\rangle \tag{208}$$

$$= \frac{1}{4} \left\langle \left| e^{2im\breve{\phi}} H \delta\tilde{X}_{C,l} - e^{-2im\breve{\phi}} H^* \delta\tilde{X}_{C,l} \right|^2 \right\rangle + \frac{1}{4} \left\langle \left| e^{2im\breve{\phi}} H \delta\tilde{Y}_{C,l} + e^{-2im\breve{\phi}} H^* \delta\tilde{Y}_{C,l} \right|^2 \right\rangle \tag{209}$$

$$= \frac{1}{4} \left| e^{2im\breve{\phi}} H - e^{-2im\breve{\phi}} H^* \right|^2 \left\langle \delta\tilde{X}^2_{C,l} \right\rangle + \frac{1}{4} \left| e^{2im\breve{\phi}} H + e^{-2im\breve{\phi}} H^* \right|^2 \left\langle \delta\tilde{Y}^2_{C,l} \right\rangle \tag{210}$$

$$= \frac{1}{4} \left| e^{2im\breve{\phi}} H - e^{-2im\breve{\phi}} H^* \right|^2 + \frac{1}{4} \left| e^{2im\breve{\phi}} H + e^{-2im\breve{\phi}} H^* \right|^2 \tag{211}$$

$$= \frac{1}{4} \left(2|H|^2 - 2|H|^2 + 2|H|^2 + 2|H|^2 \right) \tag{212}$$

$$= |H|^2 \tag{213}$$

$$= \left| -\frac{2\sqrt{\eta(1-\eta)}}{1 - i\Delta'} \right|^2 \tag{214}$$

$$= \frac{4\eta(1-\eta)}{1 + \Delta'^2} \tag{215}$$

$$\approx 4\eta(1-\eta) \tag{216}$$

where to arrive at Eq. (211) we have used the fact that the incident field entering the system through the cavity loss channels is in a vacuum state, and therefore $\left\langle \delta\tilde{X}^2_{C,l} \right\rangle = \left\langle \delta\tilde{Y}^2_{C,l} \right\rangle = 1$.

Putting this all together using Eqs. (153) and (155), we finally arrive at an expression for the variance of the measured photocurrent

$$\begin{aligned}
\left\langle |\delta i|^2 \right\rangle = {} & \frac{4\eta^2 g_0'^2 \alpha^2_{A,\text{in}}}{\left(1+\Delta'^2\right)^2} \Bigg\{ 64\eta^2 g_0'^2 \alpha^2_{A,\text{in}} \frac{\Delta'^2}{(1+\Delta'^2)^4} \left\langle g^{\text{self}\ \prime 2}_{\text{therm}} \right\rangle + \frac{16\eta^2 \alpha^2_{A,\text{in}}}{\left(1+\Delta'^2\right)^2} \left\langle R^{\text{cross}\ \prime 2}_{\text{therm}} \right\rangle \\
& + 16 g_0'^2 \alpha^2_{A,\text{in}} \left[\frac{2\eta - 1 - \Delta'^2}{(1+\Delta'^2)^2} \right]^2 \left\langle \delta\gamma_{\text{in}}'^2 \right\rangle + \frac{4\eta^2 g_0'^2}{(1+\Delta'^2)^2} \left[1 + V_{\text{RIN}}(\omega)\alpha^2_{A,\text{in}} + \frac{4\omega'^2\Delta'^2}{(1+\Delta'^2)^4} V_\zeta(\omega)\alpha^2_{A,\text{in}} \right] \\
& + \frac{4\eta(1-\eta) g_0'^2}{(1+\Delta'^2)^2} \left[1 + \frac{4\omega'^2\Delta'^2}{(1+\Delta'^2)^4} \right] + \frac{(1-2\eta)^2 + \Delta'^2}{1+\Delta'^2} + \frac{4\eta(1-\eta)}{1+\Delta'^2} \Bigg\}
\end{aligned} \tag{217}$$

$$\begin{aligned}
\approx {} & \left(\frac{2\eta g_0' \alpha_{A,\text{in}}}{1+\Delta'^2} \right)^2 \Bigg\{ \left(\frac{2\alpha_{A,\text{in}}}{1+\Delta'^2} \right)^2 \Bigg[\left(\frac{4\eta g_0' \Delta'}{1+\Delta'^2} \right)^2 \left\langle g^{\text{self}\ \prime 2}_{\text{therm}} \right\rangle + 4\eta^2 \left\langle R^{\text{cross}\ \prime 2}_{\text{therm}} \right\rangle \\
& + 4 g_0'^2 \left[\frac{2\eta - 1 - \Delta'^2}{1+\Delta'^2} \right]^2 \left\langle \delta\gamma_{\text{in}}'^2 \right\rangle + \eta^2 g_0'^2 \left[V_{\text{RIN}}(\omega) + \frac{4\omega'^2\Delta'^2}{(1+\Delta'^2)^4} V_\zeta(\omega) \right] \Bigg] + 1 \Bigg\}
\end{aligned} \tag{218}$$

$$\begin{aligned}
\approx {} & 4\eta^2 g_0'^2 \alpha^2_{A,\text{in}} \Bigg\{ 4\alpha^2_{A,\text{in}} \Bigg[16\eta^2 g_0'^2 \Delta'^2 \left\langle g^{\text{self}\ \prime 2}_{\text{therm}} \right\rangle + 4\eta^2 \left\langle R^{\text{cross}\ \prime 2}_{\text{therm}} \right\rangle \\
& + 4 g_0'^2 (2\eta - 1)^2 \left\langle \delta\gamma_{\text{in}}'^2 \right\rangle + \eta^2 g_0'^2 \left(V_{\text{RIN}}(\omega) + 4\omega'^2\Delta'^2 V_\zeta(\omega) \right) \Bigg] + 1 \Bigg\}
\end{aligned} \tag{219}$$

The uncertainty in our estimate of $g_{\rm sig}$ is then *finally* given, using Eq. (151), by

$$\left\langle\left|\delta g^{\rm est\,\prime}_{\rm sig}\right|^2\right\rangle_{\rm back-scatter} = \left(\frac{1}{64 g_0'^2\eta^4\alpha^4_{A,\rm in}\cos^2 2m(\phi_{\rm sig}-\phi_0)}\right)\left(\frac{2\eta g_0'\alpha_{A,\rm in}}{1+\Delta'^2}\right)^2\left\{\left(\frac{2\alpha_{A,\rm in}}{1+\Delta'^2}\right)^2\left[\left(\frac{4\eta g_0'\Delta'}{1+\Delta'^2}\right)^2\left\langle g^{\rm self\ \prime 2}_{\rm therm}\right\rangle + 4\eta^2\left\langle R^{\rm cross\ \prime 2}_{\rm therm}\right\rangle\right.\right.$$
$$\left.\left. +4g_0'^2\left[\frac{2\eta-1-\Delta'^2}{1+\Delta'^2}\right]^2\left\langle\delta\gamma_{\rm in}'^2\right\rangle + \eta^2 g_0'^2\left[V_{\rm RIN}(\omega)+\frac{4\omega'^2\Delta'^2}{(1+\Delta'^2)^4}V_\zeta(\omega)\right]\right]+1\right\} \tag{220}$$
$$= \left(\frac{1}{4\eta\alpha_{A,\rm in}\cos 2m(\phi_{\rm sig}-\phi_0)\left(1+\Delta'^2\right)}\right)^2\left\{\left(\frac{2\alpha_{A,\rm in}}{1+\Delta'^2}\right)^2\left[\left(\frac{4\eta g_0'\Delta'}{1+\Delta'^2}\right)^2\left\langle g^{\rm self\ \prime 2}_{\rm therm}\right\rangle + 4\eta^2\left\langle R^{\rm cross\ \prime 2}_{\rm therm}\right\rangle\right.\right.$$
$$\left.\left. +4g_0'^2\left[\frac{2\eta-1-\Delta'^2}{1+\Delta'^2}\right]^2\left\langle\delta\gamma_{\rm in}'^2\right\rangle + \eta^2 g_0'^2\left[V_{\rm RIN}(\omega)+\frac{4\omega'^2\Delta'^2}{(1+\Delta'^2)^4}V_\zeta(\omega)\right]\right]+1\right\} \tag{221}$$
$$\approx \left(\frac{1}{4\eta\alpha_{A,\rm in}\cos 2m(\phi_{\rm sig}-\phi_0)}\right)^2\left\{4\alpha^2_{A,\rm in}\left[16\eta^2 g_0'^2\Delta'^2\left\langle g^{\rm self\ \prime 2}_{\rm therm}\right\rangle + 4\eta^2\left\langle R^{\rm cross\ \prime 2}_{\rm therm}\right\rangle\right.\right.$$
$$\left.\left. +4g_0'^2\left(2\eta-1-\Delta'^2\right)^2\left\langle\delta\gamma_{\rm in}'^2\right\rangle + \eta^2 g_0'^2\left(V_{\rm RIN}(\omega)+4\omega'^2\Delta'^2 V_\zeta(\omega)\right)\right]+1\right\} \tag{222}$$

The optimum sensitivity is achieved if the molecule or nanoparticle binds at a point on the circumference of the WGM resonator where it's scattered field is exactly in, or out-of, phase with that of the intrinsic scatterer, i.e. $\phi_{\rm sig} = \phi_0 + j\pi/2$ where j is an integer. We then find

$$\left\langle\left|\delta g^{\rm est\,\prime}_{\rm sig}\right|^2\right\rangle_{\rm back-scatter} = \frac{4g_0'^2\Delta'^2}{(1+\Delta'^2)^6}\left\langle g^{\rm self\ \prime 2}_{\rm therm}\right\rangle + \frac{1}{(1+\Delta'^2)^4}\left\langle R^{\rm cross\ \prime 2}_{\rm therm}\right\rangle + g_0'^2\left[\frac{2\eta-1-\Delta'^2}{\eta(1+\Delta'^2)^3}\right]^2\left\langle\delta\gamma_{\rm in}'^2\right\rangle$$
$$+\frac{g_0'^2}{4(1+\Delta'^2)^4}V_{\rm RIN}(\omega) + \frac{g_0'^2\omega'^2\Delta'^2}{(1+\Delta'^2)^8}V_\zeta(\omega) + \frac{1}{16\eta^2\alpha^2_{A,\rm in}\left(1+\Delta'^2\right)^2} \tag{223}$$
$$\approx \frac{1}{2(1+\Delta'^2)^4}\left\langle g^{\rm self\ \prime 2}_{\rm therm}\right\rangle + g_0'^2\left[\frac{2\eta-1-\Delta'^2}{\eta(1+\Delta'^2)^3}\right]^2\left\langle\delta\gamma_{\rm in}'^2\right\rangle + \frac{g_0'^2}{4(1+\Delta'^2)^4}V_{\rm RIN}(\omega) + \frac{g_0'^2\omega'^2\Delta'^2}{(1+\Delta'^2)^8}V_\zeta(\omega)$$
$$+\frac{1}{16\eta^2\alpha^2_{A,\rm in}\left(1+\Delta'^2\right)^2} \tag{224}$$
$$\approx \left(\frac{g_0'}{2}\right)^2 V_{\rm RIN}(\omega) + g_0'^2\omega'^2\Delta'^2 V_\zeta(\omega) + g_0'^2\left(\frac{2\eta-1}{\eta}\right)^2\left\langle\delta\gamma_{\rm in}'^2\right\rangle + \frac{1}{2}\left\langle g^{\rm self\ \prime 2}_{\rm therm}\right\rangle + \frac{1}{16\eta^2\alpha^2_{A,\rm in}} \tag{225}$$

where we have used Eq. (43) to relate the self- and cross- thermorefractive noise. Comparing this to the quantum noise limit of standard single optical mode dispersive sensing given in Eq. (93) we see some striking similarities and differences. Firstly, let us examine the fundamental noise sources, shot noise and thermorefractive noise, given respectively in the last, and second last, terms in both expressions. We observe that *exactly* the same shot noise limit is reached by directly measuring back-scatter, as is achieved with a more complicated phase measurement on the single optical mode case. Furthermore, we observe that the thermorefractive noise is reduced by a factor of two. This can be understood since when thermorefractive fluctuations scatter light, only the components of the scattered field in, or out-of, phase with the intrinsic scatterer are observed. Thus, only thermorefractive noise from a smaller sample of the bulk material of the WGM resonator is sampled. The third term in both expressions results from input coupling noise, which may also be fundamental if the noise arises from thermal fluctuations in the coupling rate. Here, remembering that $\Delta' = \Delta_0' + g_0'$, we can see that again the noise contributions using one, or two, optical modes, are very similar. Here, however, detection with a single mode has an advantage, since Δ_0' may be set such that $\Delta' = 0$, in principle fully suppressing the input coupling fluctuations. In the backscatter (two mode) case, this is not possible. However, the input coupling contribution may still be perfectly suppressed by sitting exactly on critical coupling $\eta = 0.5$. The second term in both expressions is due to the frequency (or phase) noise on the light. In the one mode case, it appears that the phase noise can be removed by sitting at critical coupling, however, the reason for this is that, at critical coupling, no light (on average) exits the resonator. An optical local oscillator is then required both to boost the signal up to measurable levels and to provide a phase reference. Phase noise in this local oscillator will also introduce phase noise in the measurement. In the two mode case, direct detection is used, without the requirement of a local oscillator. Furthermore, by setting $\Delta' = 0$ the phase noise on the incident laser can be entirely suppressed. The frequency noise suppression achieved with back-scatter sensing is shown as a function

of detuning Δ' in the inset of Fig. 2 of the main paper, with a fit to the detuning dependence of the co-efficient in front of V_ζ in Eq. (224) yielding excellent agreement. Finally, the first term in both expressions is the relative intensity noise. Here the situation is reversed, relative intensity noise can be perfectly suppressed in the one mode measurement, whilst it cannot be in the backscatter measurements.

Converting to terminology consistent with the main paper, we have $\left\langle \left| \delta g_{\rm sig}^{\rm est\prime} \right|^2 \right\rangle_{\rm back-scatter} = S(\omega)$, $V_{\rm RIN} = S_{\rm RIN}$, $\omega^2 V_\zeta = S_\omega$, $V'_\gamma = S_\gamma$, $V'_{\rm therm} = S_{\rm T}/\bar{\gamma}$, and $S_{\rm shot} = \bar{\gamma}^2/16\eta^2 n_{\rm in}$ so that

$$S(\omega) = \left(\frac{g_0}{2}\right)^2 S_{\rm RIN}(\omega) + \left(\frac{g_0 \Delta}{\bar{\gamma}}\right)^2 S_\omega(\omega) + g_0^2 \left(\frac{1-2\eta}{\eta}\right)^2 S_\gamma(\omega) + \frac{1}{2} S_{\rm T}(\omega) + S_{\rm shot}, \tag{226}$$

where $S_\omega(\omega)$ is the laser frequency noise with it's relation to laser phase noise given, for example, in Ref. [2]. Furthermore, in the experiments reported in the paper the optical coupling is set to critical coupling such that $\eta = 0.5$, consequently the contribution of input coupling fluctuations is eliminated. Taking this case, and setting $\gamma = \bar{\gamma}$ we have

$$S(\omega) = \left(\frac{g_0}{2}\right)^2 S_{\rm RIN}(\omega) + \left(\frac{g_0 \Delta}{\gamma}\right)^2 S_\omega(\omega) + \frac{1}{2} S_{\rm T}(\omega) + S_{\rm shot}, \tag{227}$$

which is Eq. (1) of the paper.

[1] C. W. Gardiner and P. Zoller, *Quantum Noise: A Handbook of Markovian and Non-Markovian Quantum Stochastic Methods with Applications to Quantum Optics*, 3rd edition, Springer, 2004.

[2] J. J. Grobbelaar, *Phase noise measurement*, Masters Thesis, Stellenbosch University, South Africa (2011).

[3] A noise eater will usually work by tapping some of the laser light out of the system directly measuring it to obtain the fluctuating intensity noise, and using an amplitude modulator either in feedforward or feedback configuration to subtract this noise from the laser. In principle, with such a system, it is possible to reduce the classical noise variance such that it equals the quantum noise variance, ie $V_{\rm RIN}(\omega)\alpha_{\rm in}^2 = 1$. Commercial devices operate close to this limit in the frequency band of interest to biosensing (< 1 MHz).
